# Impact of social protection on gender equality in low‐ and middle‐income countries: A systematic review of reviews

**DOI:** 10.1002/cl2.1240

**Published:** 2022-05-25

**Authors:** Camila Perera, Shivit Bakrania, Alessandra Ipince, Zahrah Nesbitt‐Ahmed, Oluwaseun Obasola, Dominic Richardson, Jorinde Van de Scheur, Ruichuan Yu

**Affiliations:** ^1^ UNICEF Office of Research—Innocenti Florence Italy

## Abstract

**Background:**

More than half of the global population is not effectively covered by *any* type of social protection benefit and women's coverage lags behind. Most girls and boys living in low‐resource settings have no effective social protection coverage. Interest in these essential programmes in low and middle‐income settings is rising and in the context of the COVID‐19 pandemic the value of social protection for all has been undoubtedly confirmed. However, evidence on whether the impact of different social protection programmes (social assistance, social insurance and social care services and labour market programmes) differs by gender has not been consistently analysed. Evidence is needed on the structural and contextual factors that determine differential impacts. Questions remain as to whether programme outcomes vary according to intervention implementation and design.

**Objectives:**

This systematic review aims to collect, appraise, and synthesise the evidence from available systematic reviews on the differential gender impacts of social protection programmes in low and middle‐income countries. It answers the following questions:

1. What is known from systematic reviews on the gender‐differentiated impacts of social protection programmes in low and middle‐income countries?
2. What is known from systematic reviews about the factors that determine these gender‐differentiated impacts?
3. What is known from existing systematic reviews about design and implementation features of social protection programmes and their association with gender outcomes?

**Search Methods:**

We searched for published and grey literature from 19 bibliographic databases and libraries. The search techniques used were subject searching, reference list checking, citation searching and expert consultations. All searches were conducted between 10 February and 1 March 2021 to retrieve systematic reviews published within the last 10 years with no language restrictions.

**Selection Criteria:**

We included systematic reviews that synthesised evidence from qualitative, quantitative or mixed‐methods studies and analysed the outcomes of social protection programmes on women, men, girls, and boys with no age restrictions. The reviews included investigated one or more types of social protection programmes in low and middle‐income countries. We included systematic reviews that investigated the effects of social protection interventions on any outcomes within any of the following six core outcome areas of gender equality: economic security and empowerment, health, education, mental health and psychosocial wellbeing, safety and protection and voice and agency.

**Data Collection and Analysis:**

A total of 6265 records were identified. After removing duplicates, 5250 records were screened independently and simultaneously by two reviewers based on title and abstract and 298 full texts were assessed for eligibility. Another 48 records, identified through the initial scoping exercise, consultations with experts and citation searching, were also screened. The review includes 70 high to moderate quality systematic reviews, representing a total of 3289 studies from 121 countries. We extracted data on the following areas of interest: population, intervention, methodology, quality appraisal, and findings for each research question. We also extracted the pooled effect sizes of gender equality outcomes of meta‐analyses. The methodological quality of the included systematic reviews was assessed, and framework synthesis was used as the synthesis method. To estimate the degree of overlap, we created citation matrices and calculated the corrected covered area.

**Main Results:**

Most reviews examined more than one type of social protection programme. The majority investigated social assistance programmes (77%, *N* = 54), 40% (*N* = 28) examined labour market programmes, 11% (*N* = 8) focused on social insurance interventions and 9% (*N* = 6) analysed social care interventions. Health was the most researched (e.g., maternal health; 70%, *N* = 49) outcome area, followed by economic security and empowerment (e.g., savings; 39%, *N* = 27) and education (e.g., school enrolment and attendance; 24%, *N* = 17). Five key findings were consistent across intervention and outcomes areas: (1) Although pre‐existing gender differences should be considered, social protection programmes tend to report higher impacts on women and girls in comparison to men and boys; (2) Women are more likely to save, invest and share the benefits of social protection but lack of family support is a key barrier to their participation and retention in programmes; (3) Social protection programmes with explicit objectives tend to demonstrate higher effects in comparison to social protection programmes without broad objectives; (4) While no reviews point to negative impacts of social protection programmes on women or men, *adverse and unintended* outcomes have been attributed to design and implementation features. However, there are no one‐size‐fits‐all approaches to design and implementation of social protection programmes and these features need to be gender‐responsive and adapted; and (5) Direct investment in individuals and families' needs to be accompanied by efforts to strengthen health, education, and child protection systems. *Social assistance programmes* may increase labour participation, savings, investments, the utilisation of health care services and contraception use among women, school enrolment among boys and girls and school attendance among girls. They reduce unintended pregnancies among young women, risky sexual behaviour, and symptoms of sexually transmitted infections among women. *Social insurance programmes* increase the utilisation of sexual, reproductive, and maternal health services, and knowledge of reproductive health; improve changes in attitudes towards family planning; increase rates of inclusive and early initiation of breastfeeding and decrease poor physical wellbeing among mothers*. Labour market programmes* increase labour participation among women receiving benefits, savings, ownership of assets, and earning capacity among young women. They improve knowledge and attitudes towards sexually transmitted infections, increase self‐reported condom use among boys and girls, increase child nutrition and overall household dietary intake, improve subjective wellbeing among women. Evidence on the impact of *social care programmes* on gender equality outcomes is needed.

**Authors' Conclusions:**

Although effectiveness gaps remain, current programmatic interests are not matched by a rigorous evidence base demonstrating *how* to appropriately design and implement social protection interventions. Advancing current knowledge of gender‐responsive social protection entails moving beyond effectiveness studies to test packages or combinations of design and implementation features that determine the impact of these interventions on gender equality. Systematic reviews investigating the impact of social care programmes, old age pensions and parental leave on gender equality outcomes in low and middle‐income settings are needed. Voice and agency and mental health and psychosocial wellbeing remain under‐researched gender equality outcome areas.

## 1 PLAIN LANGUAGE SUMMARY

### 1.1 Social protection programmes appear to have higher impacts on women and girls than men and boys

Social protection programmes appear to have higher impacts on women and girls, who more likely than boys and men to save, invest and share the benefit from social protection programmes.

### 1.2 What is this review about?

Gender and age determine how people experience opportunities, vulnerabilities and risks. Social protection programmes, such as cash transfers, pensions and unemployment benefits aim to tackle poverty and adversity, manage risks and improve quality of life from childhood through to old age.

While social protection programmes do not negatively impact women or men, design and implementation features may lead to adverse outcomes. However, there is no one‐size‐fits‐all approach to design and implementation of social protection programmes and these features should explicitly address gender differences.

This systematic review of reviews contributes to a clearer picture of the differential impact of social protection on women and men, and girls and boys, in low‐ and middle‐income countries. It also contributes to translating this knowledge into policy actions that improve gender equality outcomes across the life‐course.

### 1.3 What studies are included?

The review includes 70 systematic reviews, representing a total of 3289 studies investigating 4 different types of social protection programmes (defined here as social assistance, social insurance, labour market and social care programmes) in 121 countries.

**What is the aim of this review?**

This systematic review of reviews summarises the evidence from 70 systematic reviews on the differential impacts of social protection programmes on women and men, and boys and girls in low‐ and middle‐income countries. The authors also reflect on implications for policy, programming, practice and research gaps arising from the evidence.

### 1.4 What are the main findings of this review?

Social assistance programmes improve labour participation, saving, investment, utilisation of health care services and contraception use among women, improve uptake of male circumcision, increase school enrolment among boys and girls and school attendance among girls. Such programmes also reduce unintended pregnancies among young women, risky sexual behaviour, and symptoms of sexually transmitted infections among women.

Social insurance programmes improve the utilisation of sexual, reproductive, and maternal health services, and knowledge of reproductive health; improve changes in attitudes towards family planning; increase uptake of male circumcision; increase rates of inclusive breastfeeding and early initiation of breastfeeding and improve physical wellbeing of mothers.

Labour market programmes improve labour participation among women receiving benefits, improve savings, ownership of assets, earning capacity among young women, and knowledge and attitudes towards sexually transmitted infections.

Labour market programmes also increase self‐reported condom use among boys and girls, increase child nutrition and overall household dietary intake, improve subjective wellbeing, economic, social and political empowerment and self‐confidence and social skills among women, and increase respect from family members in some settings.

Evidence on the impact of social care programmes on gender equality outcomes is scarce, so it was not possible to find patterns across systematic reviews.

Despite positive effects across multiple outcomes, social protection programmes with explicit objectives tend to demonstrate higher effects in comparison to social protection programmes with broad objectives.

Direct investment in individuals and families via social protection programmes must be accompanied by efforts to strengthen health, education and protection systems.

### 1.5 What do the findings of this review mean?

Important progress has been made on identifying social protection interventions that effectively address gender equality outcomes. Reviews acknowledge the crucial role of addressing gender differences in design and implementation of programmes.

There are substantial evidence gaps on the impact of social care programmes, parental leave and old age pensions on gender equality outcomes, and within the outcome areas of voice and agency, and mental health and psychosocial wellbeing.

There is a clear recognition of the potential negative impact of inadequate and unfit design and implementation features. Questions remain as to how to appropriately design and implement social protection interventions across different contexts and according to each population.

Advancing current knowledge of gender‐responsive social protection interventions requires moving beyond effectiveness studies to test packages or combinations of design and implementation features.

### 1.6 How up to date is this review?

All searches were conducted between 10 February and 1 March 2021 to retrieve all systematic reviews published within the last 10 years with no language restrictions.

## 2 BACKGROUND

Gender and age determine how people experience opportunities, vulnerabilities, and risks. In low‐income settings, adolescent girls are at higher risk of child marriage, which further hinders school enrolment and attendance, while adolescent boys are more likely to engage or be forced into child labour (Jones, 2019). During and after natural disasters, children and older adults are more vulnerable to protection harms and health risks such as poor nutrition and violence (Karunakara & Stevenson, 2012; Seddighi et al., 2017). Adult women tend to have fewer economic resources to cope with crises such as sickness or death of family members, extreme weather events or emergencies (Wenham et al., 2020) and adult men are also affected by restrictive gender norms which translate into negative social and health outcomes for all (Heise et al., 2019).

Social protection programmes, such as cash transfers, pensions, or unemployment benefits, aim to tackle poverty and adversity, manage risks, and improve quality of life from childhood through to old age. Increased socioeconomic insecurity, inadequate resources and limited access to services mean that demand for social protection is higher in low‐ and middle‐income settings. Inequality, economic insecurity and the socioeconomic shocks triggered by the COVID‐19 pandemic have widened pre‐existing gaps and further underscored the critical importance of achieving universal social protection (International Labour Organization, 2021).

Various systematic reviews point to positive effects of social protection programmes on food security (Bastagli et al., 2016), school enrolment and attendance (Baird et al., 2014), sexual and reproductive health (Owusu‐Addo et al., 2018), poverty reduction (Owusu‐Addo & Cross, 2014), access to health (Erlangga et al., 2019; Habib et al., 2016), employment (Chinen et al., 2017; Kluve, 2010) and child development (Leroy et al., 2012) in low and middle‐income countries (LMICs). Gender differences on the effectiveness of social protection programmes have been identified in some settings (Cluver et al., 2016; Gibbs et al., 2012; Manley et al., 2013). In addition, programme design and implementation may have different intended and unintended consequences for women and men at varying ages and stages of their life (Holmes & Jones, 2010).

Women's coverage of social protection programmes lags behind men's coverage (International Labour Organization, 2021). Globally 26.5% of women and 34.3% of men are legally covered by comprehensive social security systems that include a full range of benefits such as child and family benefits and old age pensions (International Labour Organization, 2021). These coverage gap can be explained by structural barriers, often associated with low labour force participation, unemployment, and informal employment (International Labour Organization, 2021). Additionally, most girls and boys still have no effective social protection coverage. According to the 2021, ILO World Social Protection Report only 26.4% of children globally receive social protection benefits, with significant regional disparities (International Labour Organization, 2021). The current evidence on the benefits and risks of social protection across gender (i.e., girls and boys, women, and men) in LMICs is yet to be consistently appraised and systematically examined. Evidence on whether the impact of different social protection programmes (i.e., social assistance, social insurance and care services and labour market programmes) differ by gender has not been synthesised and analysed. Research is needed on the contextual and structural factors that determine these differential impacts. Notably, questions remain as to whether programme outcomes vary according to intervention implementation and design. As a result, governments and organisations seeking to design, implement, de‐implement, scale up, down or close social protection programmes in LMICs face challenges when examining the evidence on social protection as a whole and its impact on gender equality indicators.

The primary aim of this review is to synthesise evidence from systematic reviews on the differential gender impacts of social protection programmes. In doing so, this review places itself at the intersection of the Sustainable Development Goals (SDGs) 1 (end poverty in all its forms everywhere) and 5 (achieve gender equality and empower all women and girls). In addition, this review informs specific targets within the rest of the SDG Agenda, such as health (target 3.8), decent work and economic growth (target 8.5) and equality (target 10.4). In the context of meeting these goals, it synthesises the evidence on social protection by gender to inform the use, design, and implementation of programmes in LMICs, contributes to building the evidence‐base of the 2030 Agenda for Sustainable Development and strengthening national initiatives for achieving gender equality and reducing poverty.

### 2.1 Description of the intervention

More than half of the global population is not effectively covered by *any* type of social protection benefit, with very low coverage in Africa (17.4%), Arab States (40%) and Asia and the Pacific (44.1%) compared to Europe and Central Asia, and the Americas (83.9% and 64.3%, respectively) (International Labour Organization, 2021). Only 44.9% of women with new‐borns receive maternity cash benefits that provide them with income security during this critical period. Just 18.6% of unemployed workers worldwide have effective coverage for unemployment and 33.5% of people with severe disabilities receive a disability benefit (International Labour Organization, 2021). Effective pension coverage for older women and men stands at 77.5% of all persons above retirement age worldwide (International Labour Organization, 2021).

However, in LMICs investment and interest in these interventions is rising. The number of LMICs with social safety nets has doubled from 72 to 149 in the last two decades (World Bank, 2017). Examples of such social protection programmes include food for education programmes (Tanzania), scholarships for low‐income families (Guatemala), electricity and fuel subsidies for low‐income households (Cambodia), and noncontributory old age pensions (Mexico).

While there is no single definition of social protection, it is hereby understood as ‘a set of policies and programmes aimed at preventing or protecting all people against poverty, vulnerability and social exclusion throughout their lifecycle, with an emphasis towards vulnerable groups’ (UNICEF, 2019; p. 2; SPIAC‐B, 2012). As such, social protection aims to both *avert* and *provide relief* from poverty and adversity (Devereux & Sabates‐Wheeler, 2004). Social protection programmes can be provided by public organisations or bodies with or without collaboration of nongovernmental organisations or private institutions. Programmes implemented solely by private organisations or nongovernmental organisations without government affiliation are hereby not considered part of social protection (UNICEF, 2019). Characteristics such as recipient, duration, frequency, and rates of social protection programme varies according to the conditions and socioeconomic disadvantages each programme aims to address. The field of social protection can be conceptualised or divided into four areas or categories (i.e., social assistance, social insurance, labour market programmes and social care) drawing from various international categorisations such as the Interagency Social Protection Assessment and UNICEF Global Social Protection Framework (SPIAC‐B, 2012; UNICEF, 2019), as defined in Table 1.

**Table 1:** Social protection categories, definitions and examples

Category	Definition	Examples
Social assistance	Cash and near cash benefits, in‐kind benefits, where receipt is not determined by individual contributions (i.e., noncontributory and publicly provided)	*For vulnerable/poor*: conditional and unconditional cash or near cash transfers. Near cash transfers such as food vouchers. Conditional and unconditional in‐kind transfers (e.g., food parcels, layettes) *For parents/caregivers/family*: childcare cash benefits/grants, birth grants, family allowances, maternity, and paternity benefits (e.g., cash benefits for pregnant and lactating women and girls, parents, parents on parental leave), death benefit, child benefit after divorce. *For income guarantee*: Universal basic income, minimum income guarantee schemes *For unemployment*: noncontributory unemployment benefits *For shelter*: housing subsidies *For old age and disability*: disability grants, social pensions, sick leave *Tax breaks for social purposes* (e.g., childcare, care for the elderly) *For encouraging access to social services* (e.g., fee waivers for healthcare, fee waivers for schooling, school vouchers, school feeding)
Social insurance	Cash or near cash benefits where eligibility is determined based on personal contributions or employer contributions (i.e., contributory schemes)	*For the parents/caregivers/family*: birth payments/benefits, maternity, paternity and parental leave, childcare cash benefits and family allowances (e.g., for public servants) *For unemployment*: unemployment benefits/insurance for former employees *For illness, injury, death*: health insurance *For shelter*: housing subsidies for employees, household contents insurance *For old age*: retirement pensions
Labour market programmes	Programmes and services that support employment and livelihoods and enable families to have enough income while ensuring provision and time for quality childcare.	*For hiring/encouraging employment*: job search programmes, hiring subsidies, wage subsidies. *Direct job creation*: public works programmes, temporary alternative employment schemes, *Skills development*: job training or skills development
Social care services	Direct outreach, case management and referral services to children and families	*For pregnancy/birth*: prenatal and post‐natal services [not primary or secondary health care, (e.g., nurse home visiting)] *For family*: family supports (e.g., parenting education, IPV interventions, centred based childcare, after school clubs) *For children and older dependents*: care for children or older people

### 2.2 How the intervention might work

**Figure 1:**
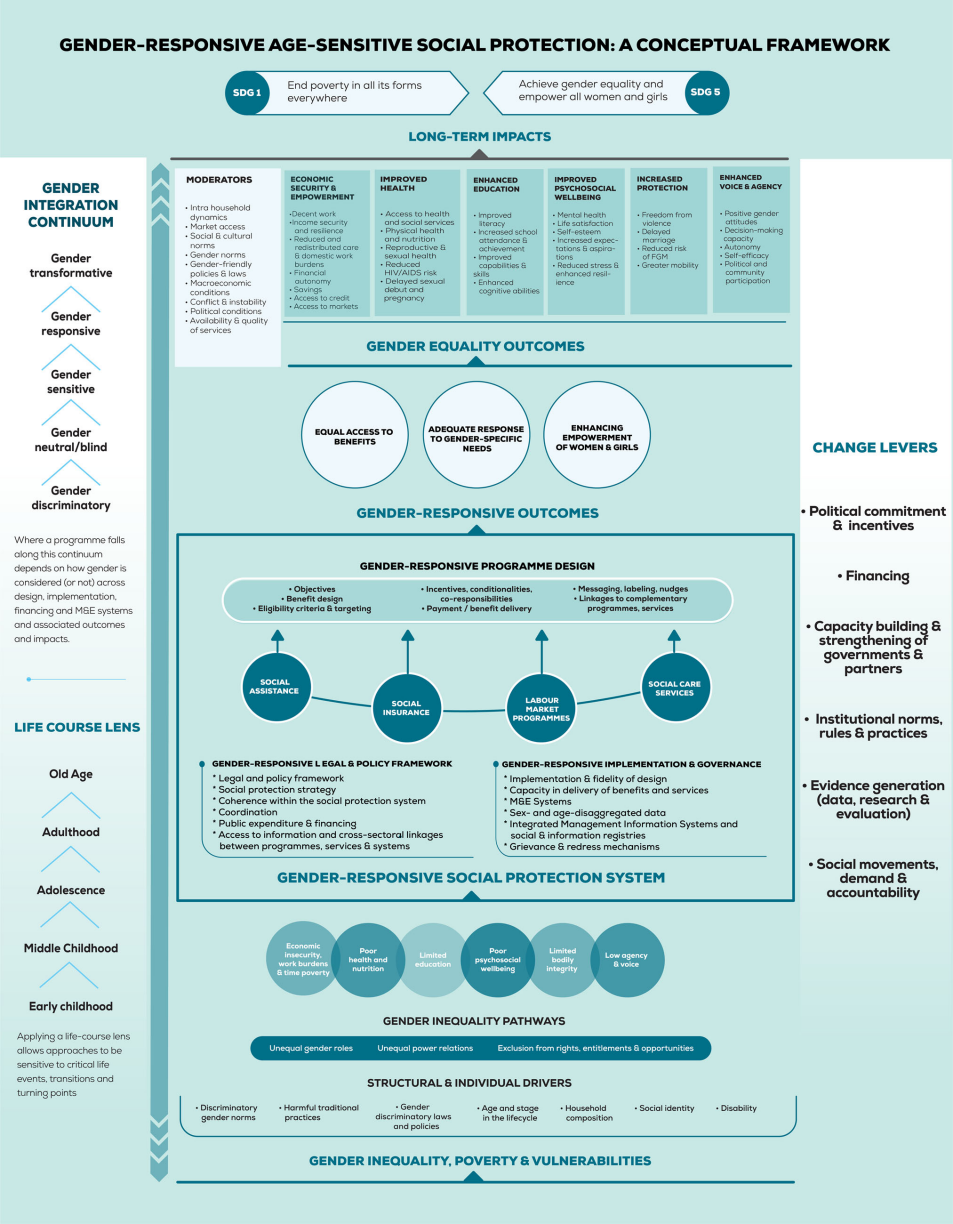
Gender‐responsive age‐sensitive social protection: a conceptual framework

The Gender‐Responsive Age‐Sensitive Social Protection Conceptual Framework (Figure 1—Reprinted with authors' permission) guides this review and delineates *how* social protection is hypothesised to lead to poverty reduction and promote long‐term and sustained gender equality (UNICEF Office of Research—Innocenti, 2020). Building on existing conceptual and theoretical efforts (Holmes & Jones, 2013), the framework starts by acknowledging that poverty and vulnerabilities are gendered, can change at different transitions and turning points throughout the life course, as well as accumulate over time. It reflects structural and individual‐level drivers of gender inequality that result in unequal outcomes for girls and women relative to boys and men, with long‐term negative impacts for them, and for sustainably reducing poverty and enhancing gender equality. It outlines moderating factors, which are dependent on context and programme design components. Integrating analysis by age and gender allows for a life course lens on gendered inequalities in relation to poverty and vulnerability.

Second, the framework maps out the opportunities and mechanisms through which social protection systems may address gendered risks and vulnerabilities through specific programmes across the social protection delivery cycle, including the legal and policy framework, programme design, implementation, governance, and financing. The conceptual framework deliberately takes a macro‐view, acknowledging the importance of a systemic and institutional perspective, beyond project or programme level pathways.

Third, the framework applies a Gender Integration Continuum (GIC), a tool to distinguish different degrees of integration of gender considerations across the social protection delivery cycle, ranging from gender‐discriminatory to gender‐transformative. The GIC helps assess the extent to which social protection systems and programmes are designed and delivered in a way that explicitly addresses gender inequality. It is based on a recognition that programmatic or policy attention to addressing gender inequality depends to a great extent on the prior understanding of prevailing gender inequalities and norms that need to be transformed through purposive actions. It thus shows how gender‐responsive social protection, by specifically addressing gendered poverty, risks, and vulnerabilities, can strengthen social protection system‐level outcomes, such as improved coverage and adequacy of social protection systems, as well as individual programme results, and thereby contribute to a range of gender equality outcomes, including economic security and empowerment, health, and education. In turn, the achievements of social protection are conceptualised to contribute to SDGs 1 and 5.

### 2.3 Why it is important to do this review

There is a large body of empirical evidence investigating the impact of social protection programmes. A myriad of robust systematic reviews have sought to clarify the impact of social protection programmes on women and men, across different age groups (e.g., Baird et al., 2014; Bassani et al., 2013; Bastagli et al., 2016; Buller et al., 2018; Chinen et al., 2017; Dickson & Bangpan, 2012; Durao et al., 2020; Haberland et al., 2018; Kalamar et al., 2016; Kluve et al., 2017; Langer et al., 2018; Målqvist et al., 2013; Murray et al., 2014; Pega et al., 2015; Tripney et al., 2013; van Hees et al., 2019; Yoong, Rabinovich and Diepeveen 2012). The results, however, are dispersed with reviews focusing on various specific sub‐types of social protection (e.g., labour market programmes, cash transfers), women and/or men, in different regions, and with some offering conflicting or discordant results regarding the impact of social protection interventions. Although various systematic reviews have gathered evidence on various areas of social protection in LMICs, evidence on the whole field is yet to be examined. For the results of a scoping exercise conducted to inform this systematic review, see the review protocol (Perera et al., 2021).

Systematic reviews summarise the best available evidence relevant to a specific research question. They are the most comprehensive way to collate all the relevant evidence on a specific topic or theme (Bakrania, 2020). The accelerated increase of systematic review publishing creates a growing interest in summarising and analysing systematic reviews. Systematic reviews of reviews help gather a wide range of evidence on interventions, enable large comparisons and can help clarify discrepant systematic review results (Polanin et al., 2017). By considering only the highest level of evidence (i.e., systematic reviews), they offer a means to review the evidence base and to obtain a clear understanding of a broad topic area (Aromataris et al., 2015). In addition, systematic reviews of reviews provide conclusions regarding research trends and gaps, making them also useful for researchers (Duvendack & Mader, 2019; Polanin et al., 2017).

A systematic review of reviews allows us to identify patterns within and across programme types and outcomes to understand *whether* and *how* social protection programmes distinctively impact women and men. This systematic review of reviews generates a clearer picture of the available evidence on the differential impact of social protection on women and men, and girls and boys, and translates this knowledge into policy actions that improve gender equality outcomes across the life‐course. As such, this review aims to inform the decisions of donors, policymakers and programme managers seeking to establish social protection programmes. More specifically, the findings of this review provide valuable insights for different components of UNICEF's and strategic partners' programmes.

## 3 OBJECTIVES

This review aims to systematically collect, appraise, map, and synthesise the evidence from systematic reviews on the differential gender impacts of social protection programmes in LMICs as well as findings on the design and implementation of these programmes. Therefore, it answers the following questions:

1. What is known from systematic reviews on the gender‐differentiated impacts of social protection programmes in LMICs?
2. What is known from systematic reviews about the factors that determine these gender‐differentiated impacts?
3. What is known from existing systematic reviews about design and implementation features of social protection programmes and their association with gender outcomes?

## 4 METHODS

### 4.1 Criteria for considering studies for this review

#### 4.1.1 Types of studies

We included systematic reviews, irrespective of publication status and the language they were published in, that synthesise and analyse evidence from qualitative, quantitative or mixed‐methods studies. As defined by the Campbell Collaboration: ‘A systematic review summarises the best available evidence on a specific question using transparent procedures to locate, evaluate, and integrate the findings of relevant research’ (Campbell Collaboration, 2014; p. 6). In addition, we adopted the following additional criteria, as outlined by Higgins & Green, 2011;

- A set of clearly stated objectives and pre‐defined eligibility criteria
- A methodology that is clearly defined allowing reproducibility
- A search strategy that allows the identification of studies meeting the pre‐defined eligibility criteria
- A quality appraisal of included studies
- A systematic synthesis, including systematic reviews that adopt a meta‐analytical, narrative, or thematic approach

Co‐registered reports were treated as duplicate reviews with data extracted from the most detailed version. Similarly, when multiple versions of the same systematic review were identified, the latest and most comprehensive version was considered for inclusion. Protocols of systematic reviews were initially included and excluded once the full review was identified. Authors were contacted when the final review was not identified to inquire whether the relevant reviews of interventions were close to completion and assess the prepublication version for inclusion in our systematic review of reviews. Other systematic reviews of reviews identified through our search were excluded.

#### 4.1.2 Types of participants

We include systematic reviews that analyse the outcomes of social protection programmes on women, men, girls, and boys in LMICs. As we are interested in the impacts of social protection during different stages of the life course, no restrictions were set on age. Studies that do not report gender‐disaggregated results of the impact of these programmes were excluded.

#### 4.1.3 Types of interventions

To be included in this review, systematic reviews had to investigate one or more types of social protection programmes. No restrictions were imposed on intervention comparison (e.g., control or waitlisted groups or regions, other interventions) to determine the relative impact of social protection interventions.

#### 4.1.4 Types of outcome measures

Our review is informed by the Gender‐Responsive Age‐Sensitive Social Protection Conceptual Framework, which establishes the following outcome areas of gender equality:

- Economic security and empowerment: Right to access opportunities and decent work, including the ability to participate equally in existing markets; control over and ownership of resources and assets (including one's own time); reduced burden of unpaid care and domestic work, and meaningful participation in economic decision‐making at all levels.
- Health: Right to live healthily, including sexual and reproductive health rights, and right to access safe, nutritious and enough food. This is also concerned with information, knowledge and awareness of health issues, and access to and expenditure on health services.
- Education: Right to inclusive and equitable quality education, leading to relevant and effective learning outcomes, including cognitive skills and knowledge; right, access to and expenditure on lifelong learning opportunities.
- Mental health and psychosocial wellbeing: A state of complete physical, mental, and social well‐being and not merely the absence of disease or infirmity, in which an individual realises their own abilities, can cope with the normal stresses of life, can work productively and is able to contribute to his or her community.
- Safety and protection: Freedom from all forms of violence (physical, sexual, and psychological violence, including controlling behaviour), exploitation, abuse, and neglect, including harmful practices (e.g., child, early and forced marriage, FGM) and child labour (including children's unpaid care and domestic work).
- Voice and agency: Ability to speak up and be heard, and to articulate one's views in a meaningful way (voice), and to make decisions about one's own life and act on them at all levels (agency).

In this systematic review of reviews, we include all systematic reviews that investigate any outcomes within any of these core areas. The use of core outcome areas has been recommended as a strategy to prevent the loss of information in systematic reviews (Saldanha et al., 2020). Narrowing down our study to a specific set of gender outcomes (e.g., increased school attendance, delayed marriage, income security) could result in missed opportunities to understand the impact of social protection on gender equality.

We report on contextual and structural factors, and programme design and implementation features determining the impact of social protection programmes. Implementation is understood as the process of fulfilling or carrying out a social protection intervention into effect (Peters et al., 2014). Intervention design or development is the period or process of developing an intervention to ‘the point where it can reasonably be expected to have worthwhile effect’ (Craig & Petticrew, 2013; p. 9) that usually consists of making decisions about the content, format and delivery and ends with the production of a document or manual describing the intervention and how it should be delivered (O'Cathain et al., 2019).

#### 4.1.5 Primary outcomes

We did not distinguish between primary or secondary outcomes, and we did not impose restrictions based on the duration of follow‐up.

#### 4.1.6 Types of settings

The reviews included in our systematic review of reviews investigate social protection programmes in LMICs, as defined by the World Bank in 2019 (Cochrane, 2020). Where systematic reviews and meta‐analyses include evidence from high‐income countries, we have only considered the findings that are presented for LMICs; we also consider systematic reviews covering regions within LMICs (e.g., Sub‐Saharan Africa). Reviews that do not disaggregate results by country, region or national income level were not included.

#### 4.1.7 Timeframe

A seminal report published in 2010 titled *Rethinking social protection using a gender lens*, identified the need to systematically appraise the evidence on social protection and gender equality (Holmes & Jones, 2010). Since the report points to the absence of systematic reviews on the field, our searches were limited to 2010 onwards.

### 4.2 Search methods for identification of studies

Our search strategy aimed to find both published and unpublished literature from a wide range of sources (i.e., bibliographic databases, institutional websites, and libraries) (Kugley et al., 2017). The search techniques used were subject searching, reference list checking, citation searching and expert consultations.

We gathered evidence from systematic reviews on the impact of these programmes on gender‐related outcomes, any determinants of these impacts as well any available evidence on the design and implementation of these interventions.

#### 4.2.1 Electronic searches

The following academic databases were searched:

- Web of Science
- Academic Search Complete (EBSCO)
- International Bibliography of the Social Science Database via ProQuest
- Africa‐Wide via EBSCOHOST
- ERIC (Education Resources Information Centre)
- Medline Complete via EBSCOHOST
- PsycINFO via EBSCOHOST
- EconLit via EBSCOHOST

In addition, a search for more reviews, especially unpublished studies and grey literature was conducted for the following institutional websites, libraries, and sources of grey literature:

- Campbell Collaboration Library
- World Bank eLibrary (https://elibrary.worldbank.org/)
- EPPI‐Centre
- IDEAS/RePEC (https://ideas.repec.org/)
- 3ieimpact evidence portal
- ILO (International Labor Organization)
- SSRN (Social Science Research Network)
- Research for Development Outputs (https://www.gov.uk/research-for-development-outputs)
- Asian Development Bank (https://www.adb.org/about/library)
- Africa Centre for Evidence—Systematic Review Repository
- Social Systems Evidence (socialsystemsevidence.org)

We ran searches in Web of Science (3,860hits), Academic Search Complete (370hits), Social Science database (39hits), Africa‐Wide via EBSCOHOST (297hits) World Bank elibrary (127hits), ERIC (50hits), Medline Complete (54hits), PsycINFO (391hits) and EconLit (188hits). The search strategies were developed using keywords and index terms (controlled vocabulary) relevant to the study concepts. Each search strategy consisted of the study concepts divided into four parts: intervention and related terms (adapted from the GRASSP Conceptual Framework—Figure 1), study design (search filter for systematic review database by 3ie), population and LMICs (adapted from Cochrane, 2020). The search strings were adapted for each database to retrieve all systematic reviews published within the last 10 years with no language restrictions. All searches were conducted between 10 February and 1 March 2021. See the review protocol for the full search strategies of academic databases (Perera et al., 2021).

#### 4.2.2 Searching other resources

This systematic review is part of a research programme investigating Gender‐Responsive Age‐Sensitive Social Protection (GRASSP) systems to enhance gender equality outcomes in low and middle‐income settings. This review is guided by the feedback and input of the GRASSP External Advisory Group (EAG). The group is composed of academics and practitioners, with expertise on social protection and gender, from UNICEF and partner organisations as well as academic institutions, including ILO, ODI, LSE and the World Bank. The EAG has provided expert advice on the subject areas of the review (i.e., gender and social protection) throughout different steps of the systematic review process. The role of the EAG members consisted of revising the search strategy, identifying systematic reviews not retrieved through the searches, providing feedback on the results of the review, as well as providing suggestions for increasing uptake and communication of findings. Experts from the EAG were consulted via e‐mail to identify systematic reviews not retrieved through the database and websites searches. In addition, reference lists of included reviews were screened to identify additional, potentially relevant, records.

### 4.3 Data collection and analysis

#### 4.3.1 Description of methods used in systematic reviews

Systematic reviews have sought to clarify the impacts of social protection programmes on gender outcomes as well as aspects of their design and implementation, using quantitative and qualitative findings from primary studies. Therefore, we adopt a broad scope to synthesise evidence from reviews investigating social protection programmes, regardless of their methodology or epistemological approach.

#### 4.3.2 Criteria for determination of independent reviews

A prevalent challenge of systematic reviews of reviews is the inclusion of systematic reviews that address similar research questions or synthesise evidence on similar and/or related interventions, which, may include some of the same underlying primary studies. The potential for ‘overlap' in primary studies between included systematic reviews introduces a risk of bias, by including the same primary study's results multiple times. As suggested by Pollock et al. (2021); in this review the degree of overlap is estimated by:

- Creating a citation matrix to visually demonstrate the percentage of overlap across each of the four intervention areas.
- Computing the Corrected Covered Area (CCA) (Pieper et al., 2014) as a measure of overlap by dividing the frequency of repeated occurrence of the index publication in other reviews by the product of index publications and reviews, reduced by the number of index publications.
- Describing the percentage of overlapping primary studies and CCA, and discussing whether and how overlap affects the results reported in the systematic review of reviews.

Briefly, the CCA is calculated with the following equation: where *N* is the sum of the number of primary studies in each review, *r* is the total number of primary studies, and *c* is the number of reviews. To assess this bias, we calculated the CCA of every two included systematic reviews in the four intervention areas, as a measure of overlap, by dividing the frequency of repeated occurrence of the index publication in other reviews by the product of index publications and reviews, reduced by the number of index publications. We listed all primary studies included in the systematic reviews and count the CCA of every two systematic reviews in four intervention areas respectively. A CCA score of less than 5% is regarded as a slight overlap, 5%–9.9% as moderate overlap, 10%–14.9% as high overlap, and over 15% as a very high level of overlap (Pieper et al., 2014).

When discussing possible overlap, is also important to consider independence from other systematic reviews of reviews. Duvendack and Mader (2019) conducted the first systematic review of reviews of the Campbell Collaboration and set a precedent for the use of the systematic review of reviews methodology to better inform the decisions of development donors, policymakers, and programme managers. This systematic review of reviews analysed the impact of financial inclusion in LMICs. Along with financial inclusion, social protection is a widely recognised and funded area of international development. Although both reviews are complementary, they constitute independent reviews. Overlaps between both systematic reviews of reviews are presented in Assessment of risk of bias in included studies.

#### 4.3.3 Selection of studies

A review author (OIO) developed the search terminology. The screening process and checklist were pilot tested at title, abstract and full text with the reviews identified through the scoping exercise. At least two review authors (CP, SB, AI, RY, JVDS) independently screened each title, abstract and full text (double‐blind screening). Disagreements were solved by consensus or by consulting another reviewer if consensus could not be reached. Two other review authors (DR, ZNA) revised the list of included reviews to confirm inclusion.

#### 4.3.4 Data extraction and management

A coding tool was developed, and pilot tested for extracting data on the following areas of interest: population, intervention, methodology, quality appraisal, findings for each research question. See the review protocol for details on each data item (Perera et al., 2021). Data from each study was extracted by four reviewers in EPPI‐Reviewer Web (Thomas et al., 2020). To ensure coding consistency, 5% of reviews were coded simultaneously by the entire team and another 10% of reviews were coded independently by two reviewers at the start of the process. Inconsistencies were solved by consensus.

##### Assessment of risk of bias in included studies

The methodological quality of the included systematic reviews was assessed by employing the Joanna Briggs Institute (JBI) Critical Appraisal Checklist for Systematic Reviews and Research Syntheses (Aromataris et al., 2015). The JBI checklist includes various considerations for the extent to which a systematic review addresses the possibility of bias in its design, conduct and analysis. These considerations include language and publication bias in the search strategy; approaches to minimising systematic errors in the conduct of the systematic review; and whether recommendations are supported by results.

The JBI Critical Appraisal Checklist has 11 criteria:

1. Is the review question clearly and explicitly stated?
2. Were the inclusion criteria appropriate for the review question?
3. Was the search strategy appropriate?
4. Were the sources and resources used to search for studies adequate?
5. Were the criteria for appraising studies appropriate?
6. Was critical appraisal conducted by two or more reviewers independently?
7. Were there methods to minimise errors in data extraction?
8. Were the methods used to combine studies appropriate?
9. Was the likelihood of publication bias assessed?
10. Were recommendations for policy and/or practice supported by the reported data?
11. Were the specific directives for new research appropriate?

Each of the questions posed in the checklist can be scored as being ‘met’, ‘not met’, ‘unclear’ or ‘not applicable’, which allows assessors to make a broad assessment of the quality of included reviews. Supporting Information Appendix 2 presents the JBI Critical Appraisal Checklist for Systematic Reviews and Research Syntheses. Reviews were given a score of 1 for each checklist criteria clearly met and 0 for those not met or unclear, with a maximum possible score of 11. Reviews scoring 8‐11 were categorised as high quality, those scoring 4–7 as moderate, and 0–3 as low‐quality systematic reviews. Reviews rated as low‐quality were excluded. To ensure consistency, two reviewers simultaneously appraised the quality of 20% of reviews at the start of the process and disagreements were solved by consensus.

##### Measures of treatment effect

As suggested by Pollock et al. (2021); we extracted and tabulated the pooled effect sizes of gender equality outcomes of meta‐analyses as reported by the review authors.

##### Unit of analysis issues

We extracted information at the systematic review level. However, when only a subset of the studies included in a review meet our inclusion criteria, data was extracted from the results that relate to said studies. To ensure that this data refers to the specific studies, extracted data was cross‐checked with the primary study. Lastly, we extracted results from systematic reviews as reported by the review authors.

##### Assessment of reporting biases

One of the items on the JBI checklist (criteria 9) assesses whether the review authors carry out an investigation of publication bias and discuss the impact this had on their review findings. Any other observations relating to other types of reporting biases (e.g., language, location, citation, outcome reporting biases) were noted and addressed in the discussion section of the review.

##### Data synthesis

This systematic review of reviews employed framework synthesis as the synthesis method. Framework synthesis is a method used in systematic reviews to examine complexity in which an a priori conceptual framework shapes the understanding and analysis of the research problem (Brunton et al., 2020). There are several reasons why this approach is suitable for this review. First, it can be applied to reviews of complex interventions and where there is a broad thematic scope (Brunton et al., 2020; Snilstveit et al., 2012). This review encompasses an entire domain of interventions (social protection), which itself comprises multiple intervention types and sub‐types. The GRASSP Conceptual Framework (Figure 1) illustrates how complex the linkages and pathways are between interventions and gender outcomes. Secondly, as Flemming et al., 2019 argue, framework synthesis allows for the juxtaposition of quantitative and qualitative evidence. Our review seeks to address not only the impacts of different social protection interventions, but also the differential impacts according to gender and age, the factors that determine those impacts and the circumstances under which the intervention might work. The GRASSP Conceptual Framework serves as a ‘scaffold’ for collating quantitative and qualitative evidence on complex social protection interventions from different types of review. The approach to framework synthesis described by Brunton et al. (2020) consists of three steps, as summarised below.

1. Framework selection and familiarisation
  Prior work undertaken within the broader GRASSP programme, in scoping the literature and developing the GRASSP Conceptual Framework constitutes part of this stage. The framework proposes a systematic, holistic, and integrated approach for conceptualising the intersections between gender and social protection. It provides this review with a typology of social protection interventions and gender equality outcome areas, as well as delineating the structural and individual drivers, moderators and design and implementation elements that may determine gender outcomes. It was developed through a review of the literature and refined through consultations with gender and social policy experts. The current framework builds on and expands existing conceptual and theoretical efforts focused on integrating a gender lens into public policy (UNICEF Office of Research—Innocenti, 2020). The scope of this review is determined by the GRASSP Conceptual Framework. This process, along with the previously described scoping exercise, contributed to the review team's familiarisation with the selected framework.
2. Indexing and charting
  The GRASSP Conceptual Framework provides a basis for searching for, screening, and extracting data from included reviews. The search strategy translates the key concepts from the typologies of interventions and outcomes. Our approach to the data, themes, and categories to be coded are driven by the way in which the interventions, outcomes, structural and individual drivers, moderators and design and implementation factors are represented in the framework. Data extraction draws directly from the typologies contained within the framework. This provides us with an initial scaffolding for grouping characteristics from each review into categories and deriving themes from this data. Framework synthesis is iterative in nature (Petticrew et al., 2013) and therefore allows for both a deductive and inductive approach to synthesis. This allows to extract and synthesise data from qualitative and quantitative reviews that may have different epistemological underpinnings, which is necessary to answer our research questions. We took a partly deductive approach to answering our research questions. We draw on reviews, including but not limited to systematic reviews of effectiveness. From these studies, we extracted data on programme impacts, and on differential impacts on gender and age sub‐groups. In this way, our synthesis of data from reviews of quantitative studies has much in common with current deductive approaches to the narrative synthesis of quantitative findings. Answering research question 2 entailed extracting data iteratively on factors that may influence the impacts of social protection programmes on gender equality outcomes, as represented in the GRASSP Conceptual Framework. Similarly, for research question 3, we built upon the typology of implementation and design issues considered in the framework. In this iterative synthesis, the results need to be organised so that patterns in findings from design and implementation of interventions can be identified across reviews (Popay et al., 2006).
3. Mapping and interpretation
  The main concepts for interventions, outcomes, contextual factors, and implementation and design issues have been identified in the GRASSP Conceptual Framework and were supplemented with additional themes emerging from the included reviews (Snilstveit et al., 2012). The first and third authors (CP and AI) synthesised the extracted data across each research question independently and then revised and merged each other's synthesis to produce a common synthesis of findings *by* outcome area (i.e., economic security and empowerment, health, education, mental health and psychosocial support, voice and agency and safety and social protection), which was discussed with and revised by the second author (SB). Following this, the two authors (CP and AI) identified and drafted key findings *across* outcome areas which were then checked and validated by the second author. All key findings were revised in collaborative discussions with all co‐authors based on the synthesis of findings by outcome area. Theories and pathways presented by the authors were also considered and were included in our analysis. When reviews offered discordant results, findings are presented along with a discussion on potential reasons for differing results. Results from meta‐analysis were included based on review authors' interpretation of their findings. However, to offer more information, we tabulated the pooled effect size of meta‐analyses that provided gender disaggregated findings (Supporting Information Appendix 6).

##### Subgroup analysis and investigation of heterogeneity

The relationships or subgroup analysis explored as part of step 2 of framework synthesis include exploring different outcomes across gender and age groups (e.g., women and men, adolescent girls and boys, older adults) to investigate differences in outcomes as well as what factors explain any identified patterns.

## 5 RESULTS

### 5.1 Description of studies

#### 5.1.1 Results of the search

A total of 6265 records were identified through 19 databases, libraries, and institutional websites, of which 1015 were duplicates. After removing duplicates, 5250 records were screened independently and simultaneously by two reviewers (AI, JVDS, RY, CP) based on title and abstract and 298 full texts were subsequently assessed for eligibility. An additional 48 records, identified through the initial scoping exercise, consultations with experts and citation searching, were also screened. After quality appraisal, 15 systematic reviews were classified as low‐quality and excluded from the review.

**Figure 2:**
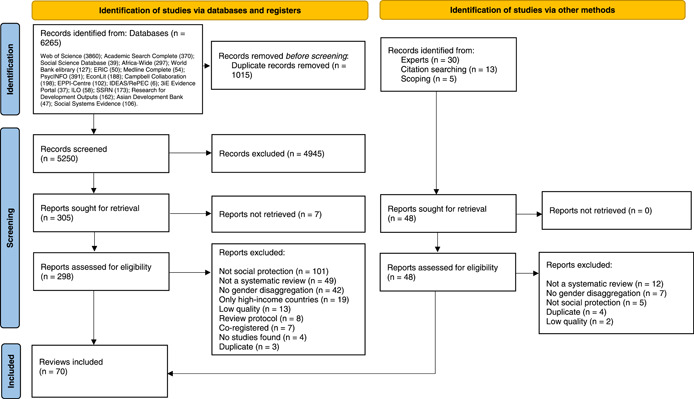
PRISMA flow diagram

Upon screening and quality appraisal completion, 70 systematic reviews, representing a total of 3289 studies, met the criteria for inclusion and were taken forward for data extraction and analysis. Figure 2 outlines the process of identifying reviews via databases, expert consultations and citation searching.

#### 5.1.2 Included studies

**Figure 3:**
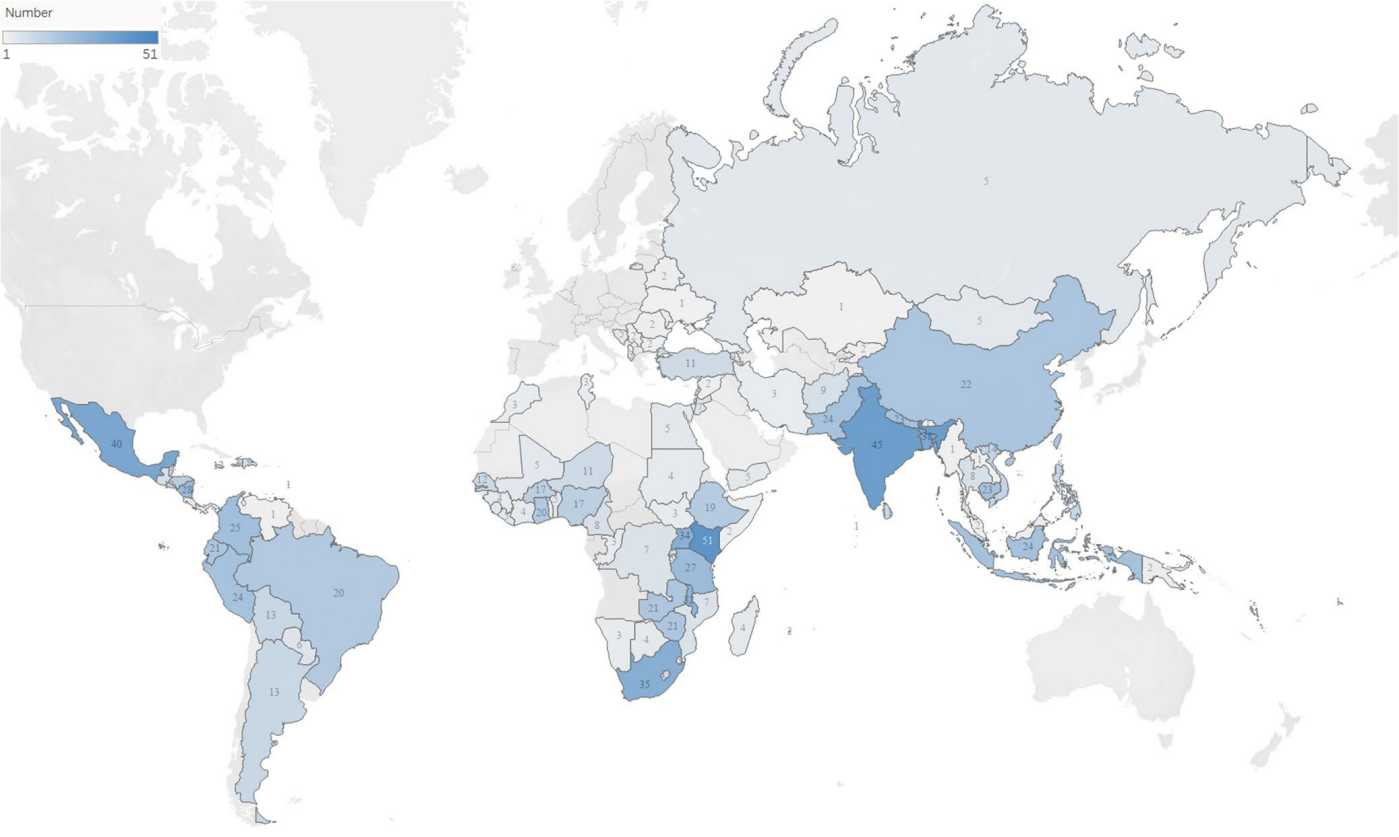
Geographical distribution of low and middle‐income countries of primary studies per review. High‐income countries included in systematic reviews' primary studies are not presented in this map

Of the 70 reviews, 9 had global geographical coverage and the remaining 61 focused on LMICs covering a total of 121 countries, with only one review specifically narrowing the scope to contexts of humanitarian emergencies. Of the 70 included systematic reviews, two focused specifically on Sub‐Saharan Africa. Figure 3 presents the geographical spread of primary studies. Kenya (*N* = 51), India (*N* = 45), Mexico (*N* = 40) and Bangladesh (*N* = 38) are the top four represented countries. Given the large number of primary studies within each systematic review, the country where the study was conducted was extracted once regardless of how many studies within the review were conducted in each country. Supporting Information Appendix 3 presents a summary of included reviews according to authors, number of included studies, type of review and analysis, geographic focus, gender and life course, intervention area and outcome area.

Reviews were published between 2011 and 2021, with most (46%, *N* = 32) being published between 2015 and 2017. The number of studies included in each review ranged between 3 and 420, with a mean value of studies of 47. However, this is mainly driven up by eight reviews with over 100 included studies each (Bastagli et al., 2016; Clifford et al., 2013; Doocy & Tappis, 2017; Kluve et al., 2017; Oya et al., 2017; Waddington et al., 2014; World Bank, 2014), with one outlier with 420 included studies (Snilstveit et al., 2015). Only one of the included reviews was published in a language other than English (Portuguese; Santos et al., 2019).

Most reviews included qualitative synthesis methodologies (87%, *N* = 61). Of these, most employed narrative synthesis (44% of the 61 qualitative analysis reviews, *N* = 27), followed by thematic analysis and descriptive synthesis (both at 10%, *N* = 6). Systematic reviews conducting meta‐analyses made up 31% (*N* = 22) of the sample and 24% (*N* = 17) of reviews employed both quantitative and qualitative methodologies to synthesise their findings. Reviews included primary studies with multiple designs, with most focusing solely on quantitative methodologies.

Most reviews (63%) did not impose any age restrictions, and 16% focused only on children (0–19 years of age). As it was part of the review's inclusion criteria, all reviews provided some sort of gender disaggregated outcomes. Most recipients of programmes were women and men, and 24% targeted women alone. Other specific populations of focus included: low‐income households, mothers, caregivers and children, women of child‐bearing age, women of working age, smallholder farmers, population affected by humanitarian crises.

Most reviews (77%, *N* = 54) investigated social assistance programmes, 40% (*N* = 28) investigated labour market programmes, 11% (*N* = 8) focused on social insurance interventions and 9% (*N* = 6) focused on social care interventions. Most of the reviews included only one intervention type (69%, *N* = 49), with 24% (*N* = 17) including two types of intervention, most of which are a combination of social assistance and labour market interventions, 26% (*N* = 18) of the total number of reviews; 6% (*N* = 4) including three or four types of intervention, and 13% (*N* = 9) including also other forms of interventions that are not social protection (e.g., microfinance, health, and education interventions). Most studies (69%, *N* = 48) included in the reviews assessed impact of the intervention in comparison to control groups (e.g., including non‐beneficiary populations or treatment as usual, beneficiaries with and without disabilities, and before and after comparisons). Just over a third of reviews analysed results of both control and other interventions (36%, *N* = 25) and 43% (*N* = 30) only included studies with a comparison condition (e.g., conditional vs. unconditional transfers, different types of transfer modalities and transfer size).

**Figure 4:**
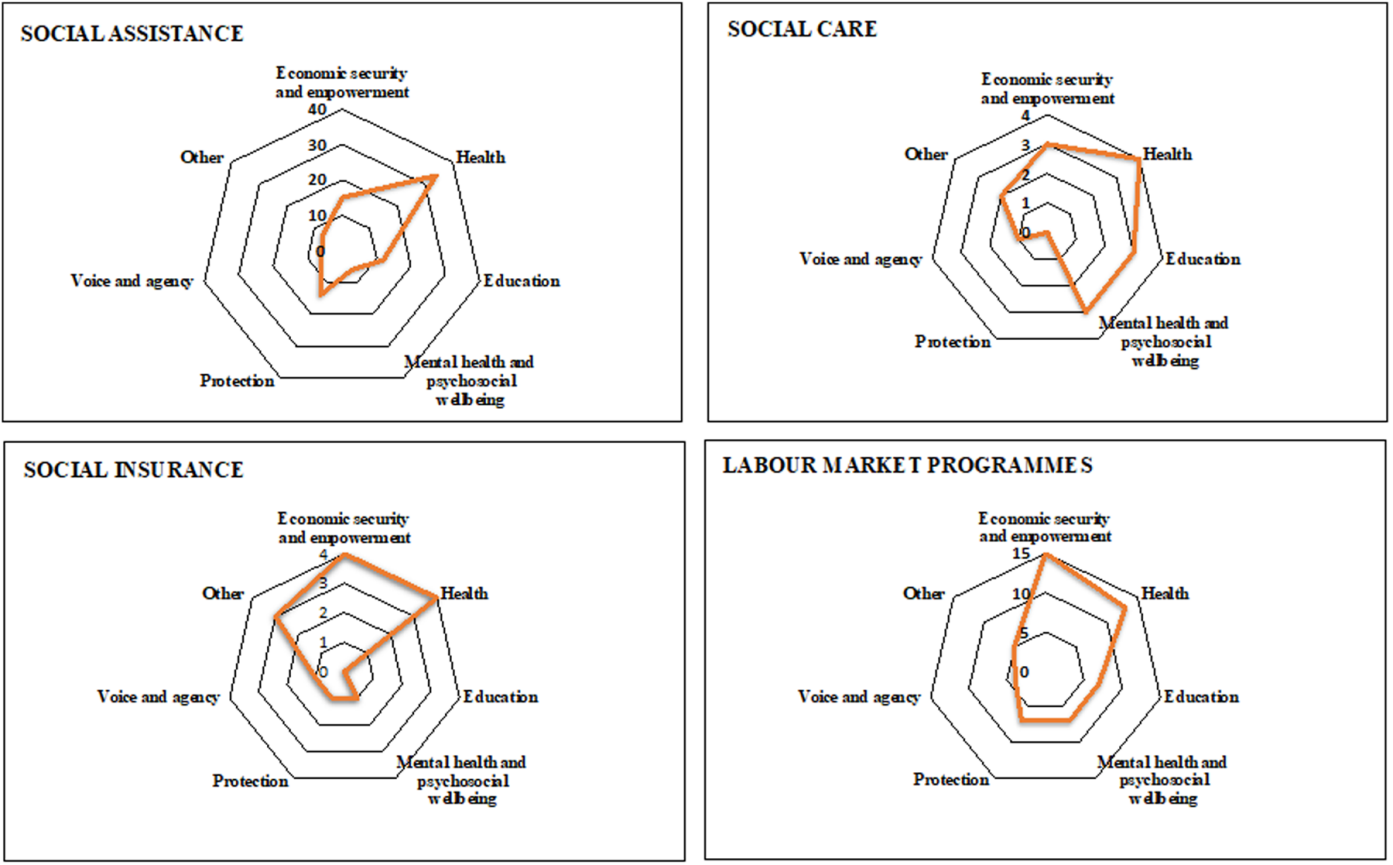
Outcome areas by intervention type

Health (e.g., utilisation of services, knowledge of sexual and reproductive health, anthropometric measures) was the most covered (70%, *N* = 49) outcome area among the 70 reviews. This is mainly driven by social assistance programmes (Figure 4). Economic security and empowerment (e.g., employment, savings, expenditure; 39%, *N* = 27) was the second most researched outcome area followed by education (e.g., school enrolment and attendance, test scores; 24%, *N* = 17). Most reviews covered a single outcome area (59%, *N* = 41) with less than half that number covering two types of outcomes areas. Economic security and empowerment and health were most frequently investigated together (e.g., nutritional outcomes and household expenditure; 26%, *N* = 18), followed by either of these with education (e.g., school enrolment and household employment, sexual and reproductive health outcomes, and school attendance; 17%, *N* = 12). Interventions were mostly provided by government agencies (73%, *N* = 51), followed by partnerships with NGOs (51%, *N* = 36) and private institutions (e.g., private health facilities) were involved in 26% (*N* = 18) of reviews. Figure 4 presents the distribution of interventions by outcome type. A list of the specific interventions and indicators considered within each review is available upon request.

#### 5.1.3 Excluded studies

During title and abstract screening, most records (63%) were excluded for not meeting the criteria of systematic review, 28% were excluded based on not reviewing social protection interventions and the remaining 9% were excluded for other reasons (e.g., focus on high‐income countries alone). At full‐text screening, most records (35%) retrieved through academic databases and institutional websites were excluded for not addressing at least one type of social protection intervention, 16% were not systematic reviews, 14% did not provide gender disaggregated results and the remaining 35% were excluded for other reasons (e.g., focus on high‐income countries alone, low‐quality, protocol of review).

### 5.2 Risk of bias in included studies

The methodological quality of included reviews was assessed using the JBI Critical Appraisal Checklist for Systematic Reviews and Research Syntheses. As explained in the review protocol (Perera et al., 2021), low‐quality reviews were excluded from this systematic review. Although these reviews may offer contributions to the study of the impact of social protection programmes on gender equality, their quality hinders the validity of their findings and conclusions and including them could have affected the overall validity of this systematic review of reviews.

**Figure 5:**
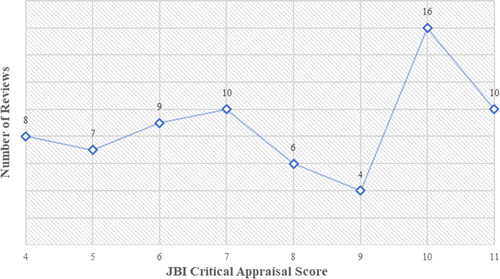
Number of reviews by JBI critical appraisal score (high‐moderate). JBI, Joanna Briggs Institute

Low‐quality reviews were excluded based on unclear or no reporting of methodological aspects such as synthesis process, appraisal of primary studies and sources and resources used to conduct the search. Upon appraisal completion, 51.4% (*N* = 36) of reviews were rated as high quality (JBI score = 8–11) and 48.6% were rated as moderate quality (JBI score = 4–7). Ten reviews received the highest quality score (Baird et al., 2013; Brody et al., 2015; Chinen et al., 2017; Doocy & Tappis, 2017; Kristjansson et al., 2015; Langer et al., 2018; Pega et al., 2015; Pega et al., 2017; Tripney et al., 2013; Waddington et al., 2014) and eight reviews received the lowest moderate quality score (Bassani et al., 2013; Buller et al., 2018; Dammert et al., 2018; Glassman et al., 2013; Halim et al., 2015; Kabeer et al., 2012; Kennedy et al., 2020; Skeen et al., 2017). Figure 5 presents the number of reviews by quality score. Supporting Information Appendix 5 presents a list of included high and moderate quality reviews and a list of reviews excluded due to low quality, as well as a graph of the number of reviews that scored positively across each JBI item.

#### 5.2.1 Independence of review—Overlap

Given that all included reviews were published in the same decade (2011–2021), it is likely that reviews overlap on different aspects of their inclusion criteria and therefore draw on the same pool of studies. We created citation matrixes to visually demonstrate the degree of overlap in percentage across every two included systematic reviews in the four intervention areas, to address the potential risk of bias that inclusion of systematic reviews that address similar research questions or related interventions, which may include some of the same underlying primary studies multiple times. The overall CCA across the 70 included reviews was 0.54% which according to Pieper et al., 4'sterpretation represents a slight overlap.

Across the 54 reviews investigating social assistance programmes, we find an overall slight overlap of 1.02%. Although the overlap is low, this category of interventions demonstrates the highest overlap of all four types of social protection interventions. In addition, within this category, there are four groups of systematic reviews that have very high CCA scores exceeding 15%. We find that the highest correlations occur between three reviews investigating male circumcision (Choko et al., 2018; Ensor et al., 2019; Kennedy et al., 2014). Three reviews on maternity care services also showed high overlapping scores (Hunter & Murray, 2017; Hunter et al., 2017; Murray et al., 2014). Four reviews on the topics of child marriage, unintended pregnancies and sexually transmitted illnesses among young people demonstrate high overlapping scores (Hindin et al., 2016; Kalamar, Bayer, et al., 2016; Kalamar, Lee‐Rife, et al., 2016; Malhotra et al., 2021). In addition, 10 additional sets of systematic reviews of which the CCA score exceeds 10%, considered as a high level of overlap. Supporting Information Appendix 4 presents matrices of systematic review with overlap.

Across the 24 reviews investigating labour market programmes, we find an overall slight overlap of 0.46%. In addition, there is one group of systematic reviews that have CCA scores exceeding 15%, including the notable overlap between reviews also presented above on the topics of child marriage and unintended pregnancies and sexually transmitted illnesses among young people (Hindin et al., 2016; Kalamar, Bayer, et al., 2016; Kalamar, Lee‐Rife, et al., 2016; Malhotra et al., 2021). Besides, the correlation scores between reviews investigating the impact of labour market programmes on women also show a high overlap, as presented in the appendices (Chinen et al., 2017; Ibanez et al., 2017; Langer et al., 2018; Yoong, Rabinovich and Diepeveen 2012).

Within the social insurance interventions or programmes, which includes eight systematic reviews, only one correlation was identified between two reviews on community‐based health insurance (Adebayo et al., 2015; Dror et al., 2016), with a very high CCA score of 16.18%. Similarly, within the social care intervention area, which includes nine systematic reviews, there was only one correlation between Ibanez et al., 2017; and Langer et al., 2018 with a slightly low CCA score of 3.77%.

Lastly, two systematic reviews (Brody et al., 2015; Kennedy et al., 2014) were included in another systematic review of reviews focusing on financial inclusion interventions (Duvendack & Mader, 2019) since they both simultaneously researched social protection and micro‐finance interventions.

### 5.3 Effects of interventions

This section presents the results of the synthesis of 70 moderate to high quality systematic reviews on the impact of social protection interventions on gender equality. First, we report five key findings that were consistent across different intervention and outcome areas (‘Findings across Outcome Areas’). Next, we outline the findings on intervention effectiveness by outcome area (i.e., economic security and empowerment, health, education, mental health and psychosocial wellbeing, voice and agency and safety and protection) (‘Findings by Outcome Area’). Supporting Information Appendix 7 summarises effectiveness findings across each intervention category and Supporting Information Appendix 6 provides a summary table of pooled effect sizes of gender equality outcomes of meta‐analyses as reported by the authors of included systematic reviews.

Within each sub‐section, we report on the contextual and structural factors as well the design and implementation features that were identified as determinants of intervention effectiveness. Although separated for ease of reference, effectiveness, contextual and structural factors and design and implementation features are intertwined. In several reviews, gender differentiated effects were partially attributed to design and implementation features and were regularly influenced by contextual and structural factors. Similarly, the influence of contextual or structural factors can be addressed via appropriate design and implementation features. The findings below stem from the framework synthesis and are stated without interpretation.

#### 5.3.1 Findings across outcome areas

##### Key finding: Social protection programmes tend to report higher impacts on women and girls in comparison to men and boys

Most reviews report higher effectiveness of social protection programmes (e.g., increased saving and investment, utilisation of health care services, school attendance) on women and girls than on men and boys. This is possibly explained by women reporting lower scores at baseline (e.g., women are more likely to be unemployed, out of school, possess lower decision‐making power within household and lower social support in the community), which most primary studies do not seem to control for. The largest effects of interventions are identified in settings with the poorest indicators and among the most vulnerable populations (e.g., lowest income areas and countries, lowest levels of education, women with heavier household workloads, women living in rural areas, child labourers) (Clifford et al., 2013; Dammert et al., 2018; Dickson & Bangpan, 2012; Kluve et al., 2017; Kristjansson et al., 2015; Manley & Slavchevska 2012; Maynard et al., 2017; Oya et al., 2017; Ton et al., 2013; Tripney et al., 2013; Waddington et al., 2014; World Bank, 2014). At the same time, these vulnerabilities (e.g., limited access to information or education, women with disabilities, older people living alone, poverty, discrimination, persons affected by humanitarian emergencies) act as barriers to uptake of social protection programmes (Brody et al., 2015; Dickson & Bangpan, 2012; Maynard et al., 2017; Oya et al., 2017).

##### Key finding: Women are more likely to *save*, *invest* and *share* the benefits of social protection but lack of family support is a key barrier

Across reviews, there are indications of structural altruistic behaviour among women participating in social protection programmes whereby women seem more likely to save and invest to off‐set future shocks (Bastagli et al., 2016; Durao et al., 2020; Hidrobo et al., 2018; Kabeer et al., 2012; Owusu‐Addo et al., 2018; Tirivayi et al., 2016; World Bank, 2014; Yoong et al., 2012). Some reviews indicate that women are also more likely to allocate transfers and income on the needs of children or other members of the household (Durao et al., 2020; Kabeer et al., 2012; Tirivayi et al., 2016; Yoong et al., 2012).

At the same time, women's uptake of social protection programmes is often contingent on the support women receive from family members (Bastagli et al., 2016; Buller et al., 2018; Chinen et al., 2017; Clifford et al., 2013; Dickson & Bangpan, 2012; Gibbs et al., 2017; Hunter & Murray, 2017; Kluve et al., 2017; Kumar et al., 2018; Murray et al., 2014; Owusu‐Addo et al., 2018; Oya et al., 2017; Waddington et al., 2014; Zuurmond et al., 2012). The uptake of health care services vouchers is determined by social and cultural attitudes (e.g., expectations to return to family home after birth, childcare expectations, partners not wanting to be labelled as poor) (Gibbs et al., 2017; Hunter & Murray, 2017; Zuurmond et al., 2012). Family pressures and responsibilities also act as a barrier to uptake in labour market programmes (Chinen et al., 2017; Clifford et al., 2013; Kluve et al., 2017; Oya et al., 2017), cash transfers (Owusu‐Addo et al., 2018) and earlier discharge from hospital among women participating in social assistance programmes for maternity services (e.g., short‐term payments to offset costs and vouchers for maternity services) (Murray et al., 2014). Indeed, a review on certification schemes for agricultural production identified higher participation of women in trainings, in matrilocal setting and settings with high rates of migration among men (Oya et al., 2017).

Domestic and childcare responsibilities are key barriers to women's participation in vocational and business training programmes and the access to income associated with participation in such trainings (Chinen et al., 2017). Transfers to women are in some contexts more acceptable within families and communities if they aim to support an activity considered within the responsibilities of women, such as child nutrition (Buller et al., 2018). Our synthesis shows that transfers that do not create excessive disruptions to household gender norms may be more acceptable. In turn, transfers that disrupt gender norms are more detrimental in highly patriarchal societies (Bastagli et al., 2016; Buller et al., 2018). This is also the case for labour market programmes implemented in contexts where women are not expected to work outside the home (Chinen et al., 2017; Gibbs et al., 2017; Oya et al., 2017). Other key barriers to uptake include gender norms relating to freedom of movement, disapproval regarding their choices, disbelief in their abilities, and limited decision‐making capacity within the household (Chinen et al., 2017; Gibbs et al., 2017; Oya et al., 2017). These key barriers, however, are context‐specific and more prevalent in patriarchal or religious countries with discriminatory formal or informal laws.

##### Key finding: Adverse and unintended outcomes are attributed to design and implementation features

No reviews report negative impacts of social protection programmes on women or men. However, there are indications of *adverse* and *unintended* outcomes attributed to design and implementation features. Targeting girls through financial incentives for education may have an unintended negative impact on boys' schooling (i.e., decreases in enrolment) (Dickson & Bangpan, 2012), old‐age pensions for persons living alone may drive older adults away from families to comply with requirements (Devereux et al., 2015), the participation of mothers in labour market programmes may lead to decreases in school attendance among their adolescent daughters who make take up more of the care and domestic work in the household (Dammert et al., 2018) and girls' mental health can be negatively impacted when conditional cash transfers become the key or main source of income (Dickson & Bangpan, 2012). Several reviews (Chinen et al., 2017; Hunter & Harrison, Portela, et al., 2017; Khan et al., 2016; Langer et al., 2018; Maynard et al., 2017; Mwaikambo et al., 2011) emphasise the importance of identifying and addressing women's local barriers to access, uptake and retention of social protection such as programme‐induced expenses (e.g., child‐care, transport, medicine and material costs). Such features, however, are not generalisable and vary largely across intervention types and settings. This, combined with the lack of evidence on design and implementation, make drawing inferences on features (e.g., eligibility criteria, gender of recipient) inappropriate. The impact of social protection programmes is largely dependent on the overarching social, cultural, political, and economic context, making it critical for programmes design and implementation to be tailored to each setting and targeted group.

##### Key finding: Social protection programmes with explicit objectives tend to demonstrate higher effects in comparison to social protection programmes without broad objectives

Reviews indicate that social protection programmes with explicit objectives tend to demonstrate higher effects on targeted outcomes (e.g., child marriage or gender norms) or closely related structural factors (e.g., child marriage via school attendance) in comparison to social protection programmes with broad objectives (e.g., reproductive health, empowerment; Chinen et al., 2017; Kalamar et al., 2016b; Malhotra et al., 2021). This has been attributed to the important role of awareness, ‘framing’ of the issue, promotional, outreach and communication strategies (e.g., providing information on eligibility criteria, available facilities, resources and services) in the implementation of social protection programmes (Hunter & Harrison, Portela, et al., 2017; Murray et al., 2014). Indeed, various reviews (Brody et al., 2015; Buller et al., 2018; Chinen et al., 2017; Kumar et al., 2018; Langer et al., 2018) identify, qualitatively and quantitatively, the added value of providing social protection interventions in combination with some form of training (e.g., life‐skills, soft‐skills, financial trainings or gender training) relating to the objectives of the benefit. This finding may be connected to conditionalities, which appear to be a determinant of programme effectiveness across two outcome areas (i.e., education and safety and protection).

##### Key finding: Strengthening social protection systems contributes to acceptability, uptake, retention, and sustainability of interventions

Lastly, various reviews (Baird et al., 2013; Blacklock et al., 2016; Glassman et al., 2013; Hunter & Murray, 2017; Hurst et al., 2015; Lee‐Rife et al., 2012; Malhotra et al., 2021; Murray et al., 2014; World Bank, 2014) identify the importance of strengthening health, protection and education systems (supply‐side) to respond to the demand created by social protection programmes. This is hypothesised to increase the acceptability of services, which in turn contribute to uptake, retention, and sustainability of social protection programmes.

#### 5.3.2 Findings by outcome area

##### Economic security and empowerment

Various social assistance and labour market programmes are associated with higher labour participation among women in comparison to men. Higher, although small, effects in overall employment, formal employment, hours in paid employment and earnings were found among women participating in vocational and business trainings in comparison to control groups. Interventions that provide job skills training with job placement services are an effective approach to increase women's wage labour participation in higher‐growth sectors in LMICs (Langer et al., 2018). Various reviews (Brody et al., 2015; Chinen et al., 2017; Langer et al., 2018) identify higher returns of social assistance and labour market programmes for women in comparison to control groups when provided in combination with some form of training (e.g., life‐skills, soft‐skills or financial trainings). However, programmes aiming to address various economic objectives by integrating *multiple* social protection programmes may require longer exposure to achieve significant results (Haberland et al., 2018). In addition, despite these positive findings on social assistance and labour market programmes, improvements across various economic outcomes (e.g., formal employment, earnings, self‐employment) may decrease over time (e.g., 6‐month follow‐up), especially when programmes are discontinued (Chinen et al., 2017; Haberland et al., 2018).

**Figure 6:**
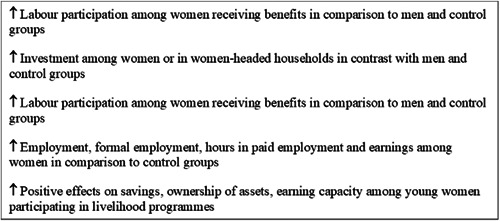
Summary of key effectiveness findings on economic security and empowerment outcomes. Upward arrow indicates an increase on an outcome attributed to a social protection intervention and downward arrow indicates a reduction on an outcome attributed to a social protection intervention

Several systematic reviews point to an association between social assistance programmes, particularly conditional or unconditional cash transfers, and significantly higher investment (e.g., savings, investment in livestock and agricultural tools) among women or in women‐headed households in contrast with men and control groups (Bastagli et al., 2016; Hidrobo et al., 2018; Owusu‐Addo et al., 2018; World Bank, 2014). Of note, while existing reviews use the terms male or female‐headed households, in this review we use the terms households headed by women or men when discussing household headship. Similar associations (i.e., positive effects on savings, ownership of assets, earning capacity) were identified among young women (10‐24 years old) participating in livelihood programmes (i.e., livelihood programmes often combine an economic transfer with a skill or knowledge component; Dickson & Bangpan, 2012). A review conducted by the World Bank, 2014 argues that this finding is consistent with evidence on altruistic behaviour identified among women, whereby they are more likely to pool resources from social assistance to offset future shocks. Figure 6 summarises key findings on the effectiveness of social protection programmes on economic security and empowerment outcomes.

###### Contextual and structural factors

Some reviews establish associations between social assistance programmes and higher intensity (e.g., number of hours) of work *outside* the home among women, increase in time spent *in* domestic work as well as a reduction in time spent in domestic work among boys and girls (Bastagli et al., 2016; Kabeer et al., 2012; Owusu‐Addo et al., 2018; Tirivayi et al., 2016; World Bank, 2014). Kabeer et al. (2012) also identify the opposite trend whereby social protection programmes encouraging school enrolment among girls, are likely to entail a reduction in their involvement in domestic chores. This can have knock‐on effects on their mothers' use of time, perhaps adding to their workloads, particularly when mothers are largely responsible for the time involved in meeting conditionalities. An increase in boys' schooling would be more likely to entail a reduction in their involvement in paid work, with implications for household income and a possible increase in fathers' time in paid work, although the reduction in income could equally be offset by drawing nonworking mothers into paid work (Kabeer et al., 2012). According to Ibanez et al. (2017), this finding is consistent with the triple burden of work paradigm, whereby women gain access to labour market opportunities through social protection programmes but continue to be responsible, or gain new responsibilities, for housework and childcare.

Women are also more likely to allocate transfers and income on the needs of children or other family members as identified by some authors (Durao et al., 2020; Kabeer et al., 2012; Tirivayi et al., 2016; Yoong et al., 2012). However, the latter expenditure pattern is not consistent across reviews. For example, a review of both conditional and unconditional cash transfers found the opposite trend whereby in contexts where men are considered breadwinners, there might also be higher expectations on them to allocate funds to childcare than if the benefit was offered to women (Owusu‐Addo et al., 2018). Expenditure patterns could be determined by a range of context‐specific factors such as gender norms, expectations, and intra‐household dynamics (Bastagli et al., 2016; Owusu‐Addo et al., 2018).

###### Design and implementation features

Design and implementation processes that are gender‐responsive and contextually and culturally fit are key determinants of programme effectiveness (Brody et al., 2015; Dickson & Bangpan, 2012; Langer et al., 2018). This entails identifying and addressing local barriers to women's participation and retention in social protection programmes by, for example, covering costs of transportation, child‐care, additional resources (e.g., legal expenses, food, construction materials) (Chinen et al., 2017; Maynard et al., 2017), providing training on *how* to use a specific benefit or navigate local financial systems such as saving accounts (Dickson & Bangpan, 2012), raising awareness on women's eligibility (Murray et al., 2014) or assisting women in safely negotiating barriers imposed by family members (e.g., disbelief regarding their abilities, mobility restrictions established by family members) (Oya et al., 2017; Waddington et al., 2014). Failure to consider contextual factors (e.g., gender norms, expenditure patterns, unequal distribution of housework responsibilities, socioeconomic background, local demand for labour) when designing and implementing social protection programmes could lead to the implementation of programmes that participants do not use, understand (Waddington et al., 2014) or need (Ton et al., 2013). They may exacerbate gender differences in vocational choices (Chinen et al., 2017), exclude the target population (Devereux et al. 2015), are not safely delivered (Doocy & Tappis, 2017) or inaccessible (Chinen et al., 2017; Oya et al., 2017).

##### Health

Most of the evidence related to the impact of social protection programmes (i.e., vouchers, cash transfers, community‐based health insurance and paid maternity leave) on health outcomes focuses on the impacts on sexual, reproductive, maternal, new‐born and child health, including service utilisation, with a few reviews focusing on the impact of these programmes on male circumcision.

Reviews provide evidence of associations between receiving cash transfers and reductions in risky sexual behaviour and increases in contraceptive use and birth spacing among women (Bastagli et al., 2016; Hindin et al., 2016; Ibanez et al., 2017; Khan et al., 2016) as well as declines in symptoms of sexually transmitted infections and sexual activity among girls participating in education‐related conditional cash transfer programmes (Kalamar et al., 2016a). Some of these changes could to be explained by women and families using the extra income to opt out of or avoid entering sexual relationships that are to some extent transactional (Bastagli et al., 2016). Associations have also been identified between life‐skills programmes and improvements in self‐reported condom use among boys and girls (Kalamar, Bayer, et al., 2016). Livelihood programmes for young women, on the other hand, do not seem to be associated with changes in sexual and reproductive health outcomes (e.g., contraceptive use) (Dickson & Bangpan, 2012) but may show increases in knowledge and improved attitudes towards sexually transmitted infections (Dickson & Bangpan, 2012). There is no evidence of increases in likelihood of pregnancy associated with cash transfers (Bastagli et al., 2016; Mwaikambo et al., 2011; World Bank, 2014) and some studies point to a reduction associated with cash transfer programmes in unintended pregnancies among young women (Hindin et al., 2016; Owusu‐Addo et al., 2018).

Reproductive health vouchers may lead to increased use of sexual and reproductive services (e.g., increase use of abortion services, cervical cancer screening), increased knowledge of contraceptives, contraceptive use and continuation and reproductive health and changes in attitudes towards family planning (i.e., wanting fewer children) (Bellows et al., 2016; Dzakpasu et al., 2014; Hunter & Murray, 2017; Meyer et al., 2011; Mwaikambo et al., 2011; Owusu‐Addo et al., 2018). In addition, both conditional cash transfers and reproductive health vouchers can improve the utilisation of maternal health services such as pre‐natal visits, skilled birth attendance or use of health facilities for birth but had no effect on probability of receiving a tetanus toxoid vaccination or postpartum visits (Dzakpasu et al., 2014; Glassman et al., 2013; Hurst et al., 2015; Målqvist et al., 2013; Murray et al., 2014; World Bank, 2014). Despite these positive findings, it is unclear from the identified systematic reviews whether or to what extent these changes in behaviour are maintained over time or sustained if vouchers are withdrawn (Glassman et al., 2013).

Several reviews reported an overall contribution of cash transfer programmes to improved child health (Khan et al., 2016; Owusu‐Addo & Cross, 2014; Pega et al., 2015; Tirivayi et al., 2016). Pega et al., 2015 found that unconditional cash transfers provided in humanitarian settings may lead to increases in proportion of children who receive vitamins, reductions in the incidence of severe acute malnutrition and large reductions in child mortality. Conditional cash transfers significantly increase preventive healthcare visits for children and reduce the incidence of diarrhoea, self‐reported illness by pregnant women and children, dietary diversity, cognitive function and development, infant mortality, anaemia and stunting (Supporting Information Appendix 4; Bassani et al., 2013; Durao et al., 2020; Khan et al., 2016; Målqvist et al., 2013; Manley and Slavchevska 2012; Owusu‐Addo & Cross, 2014; Tirivayi et al., 2016), but there were conflicting effects on immunisation (Bassani et al., 2013; Owusu‐Addo & Cross, 2014), and no effects on wasting (Durao et al., 2020). Livelihood programmes provided to women also contribute to improved child nutrition and overall household dietary intake (Durao et al., 2020). Removing or reducing fees is associated with large and significant increase in child health care visits (Bassani et al., 2013).

**Figure 7:**
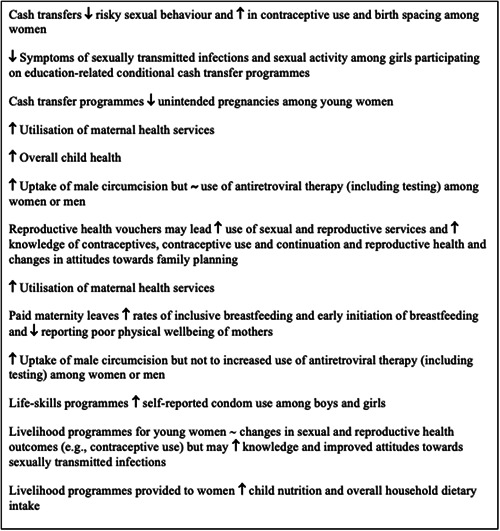
Summary of key effectiveness findings on health outcomes. Upward arrow indicates an increase on an outcome attributed to a social protection intervention; downward arrow indicates a reduction on an outcome attributed to a social protection intervention; tilde sign indicates no change on outcome attributed to a social protection intervention

Although overlaps should be noted, a range of financial incentives (e.g., cash, health service vouchers, transportation) may contribute to improved uptake of male circumcision (Supporting Information Appendix 4; Choko et al., 2018; Ensor et al., 2019; Kennedy et al., 2020) but not to increased use of antiretroviral therapy (including testing) among women or men (Choko et al., 2018). In the case of male circumcision, Kennedy et al., 2020 found that these increases, although significant, are small and could indicate that financial incentives may be acting as nudges for men that had already decided to get circumcised. Figure 7 summarises key findings on the effectiveness of social protection programmes on health outcomes.

###### Contextual and structural factors

A pattern of higher enrolment in community‐based health insurance among households headed by men has been identified in few African countries (i.e., Burkina Faso and Nigeria) (Adebayo et al., 2015), while the opposite trend was found in others (i.e., Ghana, Mali and Senegal) (Adebayo et al., 2015) and in Asia (e.g., India, China) (Dror et al., 2016). However, a review did not report any differences in the enrolment of community‐based health insurance according to gender (Van et al., 2019). Age was significantly associated with insurance enrolment, with younger women and men (ages 30–49) in Nigeria, India, Ghana, Mali, Senegal, Cameroon and Burkina Faso more likely to enrol and more willing to pay for schemes than older age groups (Adebayo et al., 2015). In addition, when it comes to incentivising male circumcision, Ensor et al. (2019) identified cash transfers as more sensitive to coercion than food or transportation vouchers.

Social and cultural attitudes towards women may act as barriers to the uptake of health vouchers (Hunter et al., 2017). Some women reported not being able to use a voucher because their husband did not want to be labelled as poor, because they were expected to return to a family home elsewhere to give birth, or because nobody was available to accompany them to a participating hospital. Of those who did travel to facility for birth care, many sought early discharge to return to look after children (Hunter & Harrison, Portela, et al., 2017).

###### Design and implementation features

A review identified an increase in maternal health service utilisation among those receiving cash or vouchers for maternity services but no reductions on stillbirth rates which might be explained by low‐quality services (Hurst et al., 2015). Social protection programmes that provide access to health care service may contribute towards increasing service demand within weak health care systems, thus exacerbating poor quality of care and deterring uptake (Bassani et al., 2013; Hurst et al., 2015; Murray et al., 2014). In turn, improving the quality of health care services, including skilled care, infrastructure and information systems, has the potential of increasing service use among participants of social protection programmes (Dzakpasu et al., 2014) and programme effectiveness (Hunter & Harrison, Portela, et al., 2017). Gaps in service quality could be met through a combination of payments for performance or investment in health facilities, medical equipment, referral systems and staff needs *and* conditional cash transfers to individuals or families (Blacklock et al., 2016; Glassman et al., 2013; Hunter & Murray, 2017; Hurst et al., 2015; Murray et al., 2014). Indeed, two reviews identify supply‐side efforts as having a major impact on maternal health service utilisation (e.g., financial provider incentives, trainings; Glassman et al., 2013; Murray et al., 2014). However, as emphasised by Hunter & Murray, 2017; these incentives need to be designed in a way that does not increase rates of medical procedures performed without medical need (e.g., unnecessary caesarean sections).

Two reviews investigated the impact of paid maternity leave on health outcomes. The benefit is associated with increase rates of inclusive breastfeeding and early initiation of breastfeeding (Carroll et al., 2020) and with a reduction in reporting poor physical wellbeing of mothers (Aitken et al., 2015). Indeed, each week increase in maternity leave was associated with a 4% reduction in the odds of mothers reporting poor physical wellbeing in Lebanon (Aitken et al., 2015).

Failure to invest in supply‐side resources could generate or exacerbate negative attitudes and behaviours from recipients towards underpaid and overworked healthcare staff, inefficient bureaucratic procedures and monitoring and procurement systems, mishandling of benefits, corruption and strain on staff and resources (Hunter & Harrison, Portela, et al., 2017; Murray et al., 2014; Waddington et al., 2014). Additional considerations of benefits aiming to increase maternal health service utilisation include the use of locally validated and inclusive eligibility criteria, collaboration and integration with local governments systems to improve monitoring and accessibility and avoid duplication and introducing measure to avoid administrative overload, extensive bureaucratic procedures and corruption (Hunter et al., 2017; Murray et al., 2014).

##### Education

**Figure 8:**
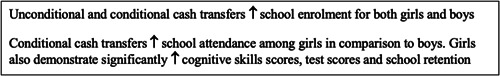
Summary of key effectiveness findings on education outcomes. Upward arrow indicates an increase on an outcome attributed to a social protection intervention; â downward arrow indicates a reduction on an outcome attributed to a social protection intervention

Unconditional and conditional cash transfers seem to contribute to improvements in school enrolment for *both* girls and boys (without difference associated with conditionality) (Supporting Information Appendix 4; Baird et al., 2013; Dickson & Bangpan, 2012; World Bank, 2014). However, the effects of social assistance programmes on child school enrolment may be higher among older children (secondary school) (Kabeer et al., 2012; World Bank, 2014). Various gender differences and considerations were identified across other education outcomes. Conditional cash transfers seem to significantly increase school attendance among girls in comparison to boys (Baird et al., 2013; Bastagli et al., 2016). Girls also demonstrate significantly higher cognitive skills scores, test scores (Bastagli et al., 2016; Dickson & Bangpan, 2012; Haberland et al., 2018; Kabeer et al., 2012) and school retention (Skeen et. al 2017) after receiving conditional cash transfers. Figure 8 summarises key findings on the effectiveness of social protection programmes on education outcomes.

###### Contextual and structural factors

Higher effects among girls' school attendance could be explained by girls being more likely to be out of school at the start of the programme (Kabeer et al., 2012). Girls may also be more likely to be engaged in domestic work and they might be able to reconcile school with their labour activities, in contrast with boys who often work outside of the home (World Bank, 2014). However, as noted by Baird et al., 2013 there is great variation across settings and context and culture may cause considerable variation in learning effect sizes.

###### Design and implementation features

Conditionality may act as a key determinant of effectiveness of cash transfers. Baird et al. (2013) found that conditional cash transfers increase the odds of a child being enroled in school by 41% and unconditional cash transfers increase the odds by 23% in comparison to control groups, without differences across girls and boys. Another design and implementation issue is noted by Dammert et al. (2018) who found that labour market programmes that incentivise women taking up economic activities may negatively impact school attendance among adolescent girls, who might be expected to carry out their mother's prior domestic responsibilities.

Targeting girls through financial incentives for education may have an unintended negative impact on boys' schooling (i.e., decreases in enrolment) (Dickson & Bangpan, 2012). Although the reasons for this gender difference are unclear, it may be explained by concerns that are specific to some contexts and not others (e.g., parents choosing to send one child to school) (Dickson & Bangpan, 2012). Similarly, affirmative action policies on scholarships, increase access to higher education among targeted groups (e.g., lower caste populations in India) but may also disadvantage young women (Clifford et al., 2013).

Lastly, various reviews note that investment in education through social protection interventions (e.g., cash transfers, vouchers) at individual and family level should be accompanied by investments in better quality education (e.g., better curricula, more accessible transportation) at school level (Baird et al., 2013; Lee‐Rife et al., 2012; Malhotra et al., 2021; World Bank, 2014).

##### Mental health and psychosocial wellbeing

**Figure 9:**
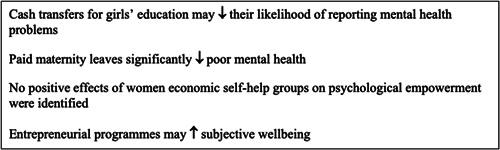
Summary of key effectiveness findings on mental health and psychosocial wellbeing outcomes. Upward arrow indicates an increase on an outcome attributed to a social protection intervention; downward arrow indicates a reduction on an outcome attributed to a social protection intervention

Included reviews provided *limited* evidence on the impact of social protection programmes on psychosocial wellbeing. A review found that 1 week's increase in paid maternity leave to be associated with a significant reduction of reporting of poor mental health (Aitken et al., 2015). No positive effects of women economic self‐help groups on psychological empowerment were identified (Brody et al., 2015). A study reviewed by Brody et al., 2015 identified adverse consequences on subjective wellbeing among women participating in self‐help groups in conservative and patriarchal contexts due to social sanctioning of women's autonomous behaviour. Although the authors do not identify average negative effects on subjective wellbeing, they caution against possible negative repercussions in these communities. In addition, Ibanez et al. (2017) did not find any overall associations between conditional cash transfers and mental health, except for entrepreneurial programmes (e.g., grants to entrepreneurs, business networks), which may be associated with small improvements on subjective wellbeing. Cash transfers for girls' education may reduce the likelihood of reporting mental health problems among young women, whether they are conditional or not (Dickson & Bangpan, 2012). Figure 9 summarises key findings on the effectiveness of social protection programmes on mental health and psychosocial wellbeing outcomes.

###### Contextual and structural factors

Due to the limited evidence under this outcome area, we were unable to identify patterns on contextual and structural factors across systematic reviews.

###### Design and implementation features

Dickson and Bangpan (2012) identified an unintended effect of cash transfer programmes. For any additional dollar offered directly to parents with conditionality on school attendance the study within the review found a negative impact on girls' mental health, possibly due to the cash becoming a key or the main source of family income and this putting pressure on girls (Baird et al., 2013; Dickson & Bangpan, 2012). Skeen et al. (2017) did not find clear patterns on specific implementation and design strategies determining the impact of interventions in improving psychosocial well‐being for children affected by HIV and AIDS.

##### Voice and agency

Evidence on the impact of social protection programmes on voice and agency was also scarce. Cash transfers programmes translate into significant improvements in women's economic decision‐making power (Owusu‐Addo et al., 2018), both as the sole and joint decision‐maker (Bastagli et al., 2016). However, there is limited evidence of any changes on women's involvement on nonfinancial decisions.

**Figure 10:**
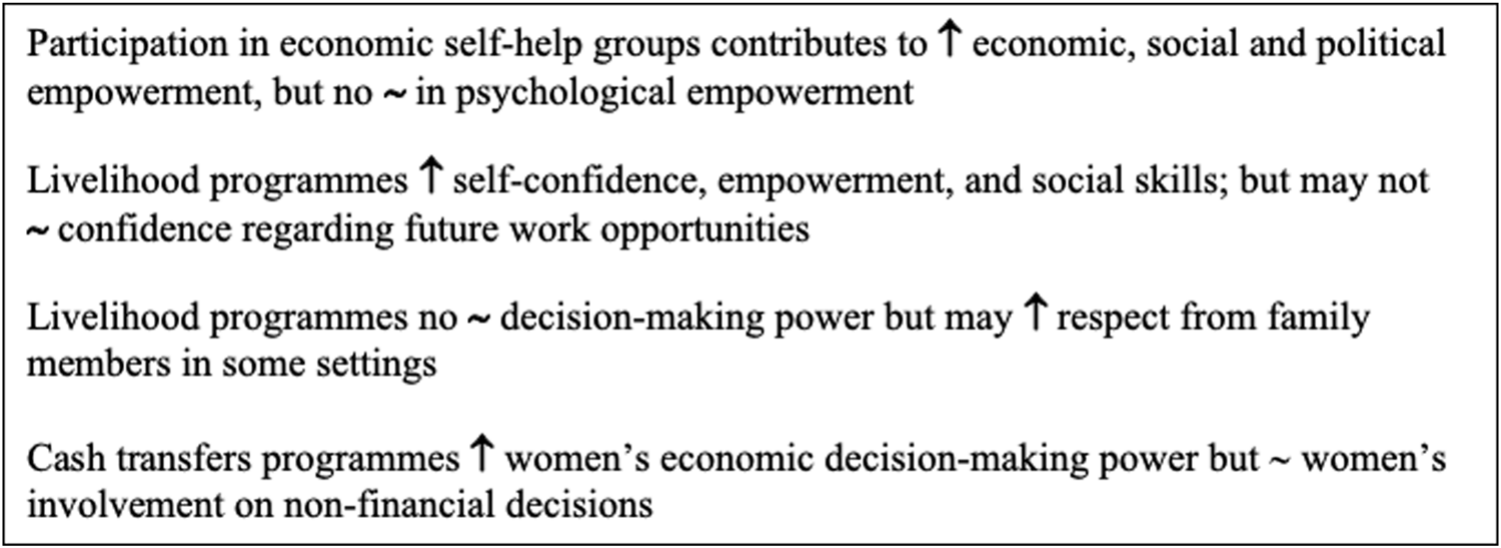
Summary of key effectiveness findings on voice and agency outcomes. Upward arrow indicates an increase on an outcome attributed to a social protection intervention; downward arrow indicates a reduction on an outcome attributed to a social protection intervention; tilde sign indicates no change on outcome attributed to a social protection intervention

Participation in economic self‐help groups contributes to economic, social and political empowerment, but not to psychological empowerment (e.g., self‐efficacy or agency, feelings of autonomy, sense of self‐worth, self‐confidence, or self‐esteem) (Brody et al., 2015). Empowerment may be stimulated by improvements in social networks, community respect, and solidarity among women self‐help group members (Brody et al., 2015). Low participation of women from the lowest socioeconomic groups has been identified in economic self‐help programmes (Brody et al., 2015). Livelihood programmes contribute to self‐confidence, empowerment, improved social skills but may not improve confidence regarding future work opportunities (Dickson & Bangpan, 2012; Haberland et al., 2018; Waddington et al., 2014). Waddington et al., 2014 found that livelihood programmes do not necessarily translate into improved decision‐making power but may lead to increase respect from family members in some settings. Figure 10 summarises key findings on the effectiveness of social protection programmes on voice and agency outcomes.

###### Contextual and structural factors

Increases in women's empowerment or their increased access to, and control over resources through social assistance programmes do not seem to ripple through the community or lead to reduced conflict, improved organisation, changes in social networks, health and community development (Ibanez et al., 2017). According to Bastagli et al. (2016) significant increases in women decision‐making power are determined by intra‐household politics, in particular gender politics, that disrupts the hypothesised linear relationship between income and power.

###### Design and implementation features

Due to the limited evidence under this outcome area, we were unable to identify patterns on design and implementation features across systematic reviews.

##### Safety and protection

Our synthesis indicated that cash transfers are consistently associated with reductions in physical abuse and sexual forms of Interpersonal Violence (IPV) but most of the evidence does not support their association with nonphysical forms of IPV (e.g., emotional, controlling behaviour)(Bastagli et al., 2016; Buller et al., 2018; Devereux et al. 2015; Gibbs et al., 2017; World Bank, 2014). Of note, controlling behaviours are sometimes conceptualised as a risk factor for IPV, rather than a type of violence itself. Bastagli et al. (2016) identified studies indicating *both* decreases and increases in nonphysical abuse associated with participation in cash transfer programmes. Buller et al. (2018) hypothesise that the overall decreased in IPV is explained by improvements in household economic security and decreases in poverty‐related and financial stress. Access to cash may reduce marital conflict that triggers violence within the household. In addition, cash transfers targeting women may empower them to exit or not engage in abusive relationships (Bastagli et al., 2016; Buller et al., 2018; Owusu‐Addo et al., 2018).

The pathways presented by Buller et al., 2018; however, do not explain the lack of impact, and in some cases increases, in rates of emotional IPV associated with cash transfer programmes. This finding could be explained by physical forms of IPV being more frequently measured in primary studies, by challenges of measuring nonphysical forms of IPV (e.g., cross‐cultural differences in measurement of emotional abuse) or using nonphysical forms of abuse such as threats to align expenditure with the men's preferences (Buller et al., 2018). According to Bastagli et al., 2016; this finding indicates that the linear relationship between income and power or autonomy within the household should always not be assumed (Bastagli et al., 2016).

Two reviews point to the effect of cash transfers in combination with other interventions (i.e., business trainings, gender and couples training, food transfers) in decreasing controlling behaviours (Bourey et al., 2015; Buller et al., 2018). Bourey et al. (2015) find that in combination with social interventions, financial incentives are associated with reductions in IPV, improved economic wellbeing, reduced acceptance of IPV, more equitable gender norms and a range of social outcomes reflecting relationship quality, empowerment, social capital, and collective action (Bourey et al., 2015). Indeed, the authors point to the potential of interventions that address economic, physical, politico‐legal, or social environments, such as social protection programmes, in contrast to individual interventions that target individual knowledge, attitudes, and behaviour to prevent IPV. Buller et al., 2018 argue that complementary interventions such as trainings and group meetings are likely to determine the impact of cash transfers on IPV through increased knowledge, self‐esteem, social interaction and capital. However, these mechanisms are seldomly explored in primary studies (Buller et al., 2018). Note that this finding should be interpreted with caution since Bourey et al. (2015); and Buller et al. (2018) present a slight overlap (4.8%) of primary studies.

Conditional cash transfers contribute to reducing remunerated and non‐remunerated child labour for both girls and boys (Bastagli et al., 2016) and mitigate economic shocks that push children into work, especially for older boys (Dammert et al., 2018).

**Figure 11:**
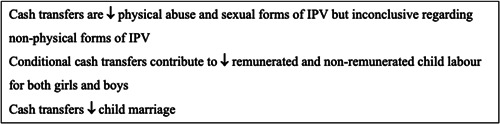
Summary of key effectiveness findings on safety and social protection outcomes. Upward arrow indicates an increase on an outcome attributed to a social protection intervention; downward arrow indicates a reduction on an outcome attributed to a social protection intervention

Owusu‐Addo et al. (2018) found that the impact of unconditional cash transfers on child marriage are not sustained over the long term in comparison to conditional cash transfers. Programmes with multiple components do not seem as effective as stand‐alone social protection programmes. This could be due to short‐term measurements of effects being more common than long term follow‐ups (e.g., 24 months after), to the higher intensity of stand‐alone programmes, lower quality of integrated interventions or by a slower uptake due to increased demands from integrated programmes on families and girls (Malhotra et al., 2021). Indeed, Lee‐Rife et al. (2012) and Malhotra et al. (2021) argue that programmes with multiple components that aim to address multiple objectives add complexity to implementation, making them harder to sustain or bring to scale. In the context of interventions aiming to tackle or prevent child marriage, educating and mobilising parents and community members as well as legal and policy efforts to change individual behaviour (e.g., reforms in minimum age of marriage, awareness raising of negative consequences of child marriage) have been identified as a key strategy to prevent this practice (Lee‐Rife et al., 2012; Petrosino et al., 2012). Figure 11 summarises key findings on the effectiveness of social protection programmes on safety and social protection outcomes.

###### Contextual and structural factors

A review indicated that a combination of increase respect of wives by husbands, increased self‐confidence, improved economic stability, exposure to gender trainings and support from other participants may explain reductions in domestic violence associated with participating in economic self‐help programmes (Brody et al., 2015). Cash can increase women's bargaining power, strengthen their self‐worth, and potentially increase their status within the household. However, the size of the transfer should be considered in relation to the context. Although the evidence is scarce, larger transfers could be associated with an increased likelihood of abuse towards women (Bastagli et al., 2016) and smaller transfers may be directed to every‐day household consumption and therefore more likely to be managed by women (Buller et al., 2018).

###### Design and implementation features

Social assistance programmes (e.g., cash or in‐kind transfers, school vouchers) and labour market programmes (e.g., life skills trainings) that emphasise on its focus on reducing child marriage in some way (e.g., through conditionality in delaying marriage, communication or through gender trainings) seem to be consistently associated with delayed age in marriage of girls; with programmes without conditionality or gender focus showing less impact (Dickson & Bangpan, 2012; Haberland et al., 2018; Kalamar et al., 2016b; Malhotra et al., 2021; Owusu‐Addo et al., 2018). Communicating the objective of the benefit seems to be a key determinant of the effectiveness of social protection interventions that aim to reduce child marriage. This is evidenced in Kalamar, Lee‐Rife, et al. (2016) and Malhotra et al. (2021) which find interventions yield better outcomes when they are communicated as focusing on child marriage than presenting a more general approach to sexual and reproductive health.

## 6 DISCUSSION

### 6.1 Summary of main results

Five key findings were consistent across intervention and outcomes areas. Although women's coverage of social protection lags behind men and most boys and girls are not effectively covered by social protection programmes (International Labour Organization, 2021), our review found that social protection programmes demonstrate higher impacts on women and girls. The largest effects of interventions are identified in settings with the poorest indicators and among the most vulnerable populations (e.g., lowest income areas and countries, lowest levels of education, women with heavier household workloads, women living in rural areas, child labourers).

Women are more likely to save, invest and share the benefits of social protection but lack of family support is a key barrier to their participation. Social protection programmes with explicit objectives tend to demonstrate higher effects in comparison to social protection programmes with broad objectives. While no reviews point to linear negative impacts of social protection programmes on women or men, *adverse* and *unintended* outcomes have been attributed to design and implementation features. However, there are no one‐size‐fits‐all approaches to design and implementation of social protection programmes and these features need to be contextually adapted within each setting and population. Direct investment in individuals and families via social protection programmes needs to be accompanied by efforts to strengthen health, education and protection systems. This will not only respond to the demand created by social protection programmes but also increase in the acceptability of services which in turn may contributes to uptake, retention and sustainability of social protection interventions.

*Social assistance programmes* improve labour participation, saving, investment, utilisation of health care services and contraception use among women, improve uptake of male circumcision, increase school enrolment among boys and girls and school attendance among girls; reduce unintended pregnancies among young women, risky sexual behaviour among women and symptoms of sexually transmitted infections. *Social insurance programmes* improve the utilisation of sexual, reproductive, and maternal health services, and knowledge of reproductive health; improve changes in attitudes towards family planning; increase uptake of male circumcision; increase rates of inclusive breastfeeding and early initiation of breastfeeding; decrease the reporting poor physical wellbeing of mothers. *Labour market programmes* improve labour participation among women receiving benefits, improve savings, ownership of assets, earning capacity among young women, improve knowledge and attitudes towards sexually transmitted infections, increase self‐reported condom use among boys and girls, increase child nutrition and overall household dietary intake, improve subjective wellbeing, improve economic, social and political empowerment and self‐confidence and social skills among women, and increase respect from family members in some settings. Only four reviews found no changes of social protection interventions on gender equality outcomes: livelihood programmes were *not* associated with changes in decision‐making power, confidence regarding future work opportunities among women or changes in sexual and reproductive health outcomes. In addition, financial incentives were *not* associated with changes in use of antiretroviral therapy (including testing) among women or men. Evidence on the impact of *social care programmes* on gender equality outcomes is scarce, which impeded finding patterns across systematic reviews.

### 6.2 Overall completeness and applicability of evidence

The 70 systematic reviews identified through this review show that significant progress has been made on identifying social protection interventions that effectively address gender equality outcomes. However, definite gaps remain. Evidence on the impact of social care programmes on gender equality outcomes is scarce, which impeded finding patterns across systematic reviews within this programmatic area. This is a key critical gap, considering that most care of children and older dependents is disproportionally undertaken by women and girls and given the disproportionate impact this has on women's economic empowerment (International Labour Organization, 2018). A few reviews investigated the impact of maternity leave on health outcomes, but no reviews investigated the impact on paid maternity leave across other outcome areas. Despite the known role of parental leave in increasing women's participation in the labour market and reducing pay gaps (Rocha, 2021), no reviews investigated the impact of paternity or parental leave on gender equality outcomes. Although old age pensions are the most common form of social protection globally (International Labour Organization, 2021), it is one the least researched areas in terms of their impact gender equality outcomes.

Gaps were also found across two outcome areas: voice and agency, and mental health and psychosocial wellbeing. Quality social protection schemes are considered a key reason for the relatively high levels of subjective wellbeing in Nordic countries (Martela et al., 2020). However, evidence on the impact of social protection programmes on mental health and psychosocial wellbeing among women and men in low and middle‐income countries is lacking. Similarly, though women and girls' empowerment and capacity to make decisions about their lives free from violence and discriminations are intrinsically linked to gender equality, we identified limited evidence for voice and agency. One possible explanation for this, is that voice and agency are mainstreamed through all other outcome areas. Another likely explanation is that changes in social norms, including harmful gender norms, occur over time while the impact of most social protection programmes is measured over relatively short periods of time. Therefore, more longitudinal assessments might be needed to ascertain impacts on voice and agency.

Overall, most reviews found that women tend to obtain increased benefits from social protection programmes. However, exposure varies largely within and across social protection programmes and it is unclear whether higher size or longer duration benefits are associated with improved outcomes over time. In addition, differences in the impact of social protection programmes between women and men are often attributed to lower baseline scores. Notably, controlling for baseline characteristics in primary studies would generate findings on which groups of women benefit the least (e.g., according to age, employment status, income level), but also whether targeting women or specific groups specifically is associated with improved outcomes. Most analyses of gender differences tend to be simple, focusing on differences between women and men or women and control groups without inquiring into sub‐group differences. As a result, and despite their importance, most reviews do not provide age‐disaggregated results or sub‐group analyses. Most reviews within our review identified social assistance programmes tend to demonstrate higher impacts on women in comparison with men. Though this is probably due to lower baseline characteristics, a caveat is that although women lag behind in social protection coverage (International Labour Organization, 2021), they are often the main or sole target of cash transfers and in some cases labour market programmes (Kluve et al., 2017). As these programmes constitute a large part of the evidence, they may be driving these differential impacts. In addition, while various reviews points to increased effectiveness of conditional cash transfers on various educational outcomes among girls as well as to overall child health, it is unclear from reviews what these conditionalities entail (e.g., soft or stricter conditionalities). In addition, such positive effects should not be *de facto* linked to conditionality as there might be contextual confounding factors that are not considered, such as transfer recipient, supply‐side constraints or self‐selection of persons that are already meeting the conditionality being more likely register and take up the benefit (Yoong, Rabinovich and Diepeveen 2012).

Consistent with previous evidence, our review found that women's access social protection is often associated with increased investments in family welfare, including children's health and education (Kabeer, 2020). However, directly targeting women as social protection beneficiaries for the explicit purpose of increasing household welfare, without addressing their ability to make and influence decisions in the household, may have unintended consequences (Camilletti et al., forthcoming). Targeting resources to women rather than men, based on the notion of their higher likelihood to invest in the education and health of children could in fact reinforce the stereotype of women as primary caregivers, further entrenching normative divisions of labour, rather than assisting in challenging them (Bastagli et al., 2016; Camilletti et al., forthcoming).

Most included reviews acknowledge the importance of design and implementation and highlight how *careful* and *appropriate* design and implementation determines programme effectiveness. Although this inconclusive finding is probably explained by how contextual these features are, reviews rarely define what constitutes careful design and implementation, how to appropriately design and implement interventions, or measure whether they are have been carefully and appropriately implemented. There is a clear recognition of the potential negative impact of inadequate and unfit design and implementation features, but more attention needs to be paid to processes for appropriately designing and implementing social protection interventions across different contexts. Given the importance of these processes, advancing current knowledge of gender‐responsive social protection entails moving beyond effectiveness studies to empirically test packages or combinations of design and implementation features that determine the impact of these interventions on gender equality.

#### 6.2.1 Conceptual framework

This systematic review of reviews employed framework synthesis to identify differential gender impacts of social protection programmes in LMICs, contextual and structural factors as well as findings on design and implementation of these programmes. The findings of this review offer some insights into the composition of the GRASSP Conceptual Framework that guided our research.

First, although conceptually separated, the available evidence does not clearly distinguish or separate structural or contextual factors from design and implementation features. Instead, those factors are interdependent. For example, while context‐specific gender norms can hinder women's participation in social protection programmes (e.g., due to childcare responsibilities, limited decision‐making capacity in the household), programmatic efforts to provide care for children or increase acceptability of social protection programmes within households act as implementation features. Similarly, structural, or contextual factors determine the impact of interventions. For example, household composition (i.e., household headed by women or men) in some context is associated with significantly higher enrolment in community‐based health insurance schemes. Therefore, while conceptually it is possible to index these constructs separately, in practice the interplay between the concepts and the evidence does not create an explicit separation. This does not suggest a reframing of the conceptual framework but instead a consideration of how current research in social protection is presented.

Second, the findings of our review indicate women and girls, especially the most vulnerable groups (e.g., lowest levels of education, women with heavier household workloads, women living in rural areas), tend to report the highest benefits from social protection programmes in comparison to boys and men across all outcome areas. One of the key features of the GRASSP Conceptual Framework is the Gender Integration Continuum, a tool to distinguish different degrees of integration of gender considerations across the social protection delivery cycle, ranging from gender‐discriminatory to gender‐transformative. Our findings support the use of measures and tools to identify gender considerations that can determine the implementation of social protection programmes. With the caveat that, the level of gender integration of a programme should not be equated with positive results among women since we find that programmes that are highly disruptive of harmful gender norms could at best lead to low uptake and at worst cause harm (e.g., aggravate harmful household dynamics).

Gender norms refer to social and cultural attitudes and expectations and are reinforced by unequal distribution of resources. To design and implement interventions that are gender responsive, even transformative, it is fundamental to understand prevailing gender inequality and context‐specific social norms, so that they can be transformed through purposive actions. No design and implementation should be undertaken without taking to account context‐specific gender norms and attitudes. Despite this, reviews do not report on methodologies or step‐by‐step processes to systematically gather gender information and adapt social protection programmes through participatory approaches across different contexts. This lack of systematic adaptation can result in missed opportunities for interventions to adequately address gender inequalities. As stated by Murray et al. (2014, p. 12) ‘success in initiating, sustaining, and scaling‐up schemes is highly dependent on a good understanding of what works in that context’.

### 6.3 Potential biases in the review process

Systematic reviews of reviews are a relatively new approach to evidence synthesis that aim to survey the evidence base to identify areas of evidence gaps and broader areas of consensus on a field of intervention. Despite its advantages, the relatively novel methodology suffers from a lack of clear guidance and is yet to develop strategies to address the challenges of processing and analysing synthesised reviews. A number of systematic reviews of reviews were consulted for methodological guidance, largely drawing from Duvendack and Mader (2019); and Polanin et al. (2017). Despite their added value, below we describe several methodological limitations of review process.

Key challenges of systematic reviews of reviews relate to external validity and attribution of effect. Some findings may be related exclusively to one study within a systematic review, which affects the external validity of a specific finding within a review. To address this gap, we encourage systematic reviewers to point to findings from single reviews and reflect on how generalisable their findings are. A related limitation is establishing causality or whether the intervention or a specific demographic characteristic of a population explains a change in a specific outcome. There are multiple variables that could determine the association between a given programme and the identified effect that might not have been measured within primary studies or not adequately reported in a systematic review.

Although adopting a broad thematic scope has allowed us to investigate gender differentiated impact across social protection programmes, the diverse range of included outcomes and interventions mean that in some cases findings, especially within the topic of design and implementation, come from a small sample of reviews or sometimes a single systematic review. Similarly, we identified five key findings that are applicable across more than one intervention area, and some apply to certain areas more than others. As noted in the analysis, we found limited evidence on old age pensions, parental leave and social care which means that, although the scope of this review is broad and includes these types of interventions, findings speak mostly to social assistance, labour market programmes and social health insurance. Despite the limited evidence on certain areas of social protection, the breadth of literature and intervention areas and outcomes remains ambitious, making it challenging to draw in depth conclusions for specific programme areas.

Synthesising evidence from an already synthesised product, makes it challenging to identify highly contextual specific design and implementation factors, unless the authors' specifically set out to analyse these features. This is further hindered by lack of adequate descriptions of interventions as part of the synthesis process, whereby authors have included a number of interventions in their review—though broadly within the same category of intervention—overlook design and implementation features when presenting results to these to specific outcomes, an issue found in other systematic reviews of review (Ekeland et al., 2010; Mikton & Butchart, 2009). As indicated in the Methods' section, we extracted results as reported within each systematic review. Most reviews do not provide definitions of each outcomes area or indicator under study. We have addressed this limitation by presenting results across the broad outcome areas of our Conceptual Framework.

Lastly, most systematic reviews present positive findings associated with receiving social protection interventions. Although through item 9 of the JBI Critical Appraisal Checklist we assessed whether an investigation of publication bias was carried out, publication bias cannot be discarded. Primary studies showing positive significant results are more likely to be published than studies with null, inconclusive or negative outcomes. Withholding negative results from publication can have damaging effects on the integrity of knowledge and on practice and is therefore a limitation of this review (Joober et al., 2012).

### 6.4 Agreements and disagreements with other studies or reviews

We do not identify any conflicts with other systematic reviews of reviews.

## 7 AUTHORS' CONCLUSIONS

### 7.1 Implications for practice

This review uncovers a series of contradictions regarding the impact of social protection on gender equality outcomes. Although social protection has increased impact on women and girls and even though women share the benefits and invests the most, their coverage lags behind and persistent inequalities within and outside the household *hinder* their access to social protection. Support from family and community members is key to increasing uptake and participation of women in social protection interventions. This entails identifying and addressing local barriers to women's participation and retention in social protection programmes by, for example, covering costs of transportation, providing training on how to use benefits or navigate local systems, raising awareness on women's eligibility or assisting women in safely negotiating barriers imposed by family members. Sustained investment in affordable and good‐quality childcare programmes could support women's participation and retention in social protection. Interventions are more effective when specific outcomes are purposely addressed, and this can be done by incorporating awareness activities and further complemented with relevant training.

There are no one‐size‐fits‐all approaches to design and implementation of social protection programmes and these features need to be adapted and take into consideration gender norms that can hinder women's participation and uptake in social protection programmes. Social protection efforts should first and foremost avoid harm. Failure to be gender‐responsive and consider factors such as gender norms, expenditure patterns, unequal distribution of housework responsibilities, socioeconomic background and local demand for labour when designing and implementing social protection programmes could lead to the implementation of programmes that participants do not use, understand or need, or negatively impact their lives by reinforcing traditional gender roles, imposing additional unpaid work on women, excluding the target population, and are not safely delivered or inaccessible.

The COVID‐19 pandemic has exacerbated pre‐existing gender inequalities (e.g., higher unemployment, unpaid care work, domestic violence, high representation in sectors hardest hit sectors among women) and the global social protection response has been largely gender‐blind (International Labour Office, 2021). This review evidence the importance of gender‐responsive social protection programmes along with the importance of system strengthening that responds to the demand for such programmes.

### 7.2 Implications for research

Although effectiveness gaps remain, current policy and programmatic interests are not matched by a rigorous evidence base demonstrating *how* to appropriately design and implement social protection interventions. Advancing current knowledge of gender‐responsive social protection entails moving beyond effectiveness studies to test packages or combinations of design and implementation strategies that determine the impact of these interventions on gender equality.

Systematic reviews are needed on the impact of social care programmes, old age pensions and parental leave on gender equality outcomes. Voice and agency and mental health and psychosocial wellbeing are insufficiently researched gender equality outcome areas of social protection programmes. In addition, we did not identify any systematic reviews investigating the impact of social insurance on education outcomes. Controlling for baseline outcomes is recommended in analyses of primary studies to ascertain sub‐group differences on the impact of social protection programmes.

## CONTRIBUTIONS OF AUTHORS

- Content: Camila Perera, Shivit Bakrania, Alessandra Ipince, Zahrah Nesbitt‐Ahmed and Dominic Richardson
- Systematic review methods: Camila Perera, Shivit Bakrania and Alessandra Ipince
- Information retrieval: Oluwaseun Ireti Obasola, Camila Perera, Shivit Bakrania and Alessandra Ipince
- Screening and data extraction: Camila Perera, Shivit Bakrania, Alessandra Ipince, Ruichuan Yu and Jorinde van de Scheur
- Data analysis: Camila Perera, Shivit Bakrania and Alessandra Ipince
- Overlap analysis: Ruichuan Yu

## DECLARATIONS OF INTEREST

None known.

## PLANS FOR UPDATING THIS REVIEW

Systematic reviews of reviews are generally updated between 3 and 5 years depending on the need of an update (availability of new reviews). Regular updates are also subject to availability of funding. If funding is available, UNICEF Office of Research—Innocenti takes responsibility for updating the review.

## DIFFERENCES BETWEEN PROTOCOL AND REVIEW

Our review made six deviations from the protocol. First, disagreements in the selection of reviews were solved by consensus instead of consultations with a third author. We considered this approach to be more suitable to the available resources and analogous to third author consultations. Second, due to the large volume of reviews meeting inclusion criteria, two reviewers simultaneously appraised the quality of 20% of reviews, instead of all reviews as originally planned. This was done at the start of the process to ensure consistency and disagreements were solved by consensus. Third, four reviewers instead of two worked on data extraction. To ensure coding consistency, 5% of reviews were coded simultaneously by the entire team and another 10% of reviews were coded independently by two reviewers at the start of the process. Inconsistencies were solved by consensus. In addition, three categories were removed from the data extraction framework (i.e., duration, intervention name and measurements) as they were not commonly reported by authors or deemed useful for answering the review's research questions. We planned to contact review authors when data missing or insufficiently reported. However, this was not feasible due to the large volume of reviews that met our inclusion criteria. Instead, we noted gaps in coverage throughout the results. Lastly, we planned to adopt the PRIO‐harms reporting checklist (Bougioukas et al., 2018) but replaced it with the PRISMA checklist (Supporting Information) due to the PRIO‐harms checklist's focus on reviews of health care interventions.

## PUBLISHED NOTES

### Characteristics of studies

#### Characteristics of included studies

Adebayo et al., 2015

**Table:** 

Methods	Systematic Review/Narrative Synthesis
Participants	No restrictions
Interventions	Social insurance
Outcomes	Health
Notes	

Risk of bias table

Aitken et al., 2015

**Table:** 

Methods	Systematic Review
Participants	Working age women
Interventions	Labour market
Outcomes	Mental health and psychosocial wellbeing, Health
Notes	

Risk of bias table

Baird et al., 2013

**Table:** 

Methods	Systematic Review/Meta‐analysis
Participants	Low‐income household with school aged children
Interventions	Social assistance
Outcomes	Education
Notes	

Risk of bias table

Banks et al., 2017

**Table:** 

Methods	Systematic Review/Qualitative Analysis
Participants	Persons with disabilities, no age restrictions
Interventions	Social assistance
	Social insurance
Outcomes	Others
Notes	

Risk of bias table

Bassani et al., 2013

**Table:** 

Methods	Systematic Review/Qualitative Analysis
Participants	Children under age 6
Interventions	Social assistance
Outcomes	Health
Notes	

Risk of bias table

Bastagli et al., 2016

**Table:** 

Methods	Systematic Review/Narrative Synthesis
Participants	No restrictions
Interventions	Social assistance
Outcomes	Economic security and empowerment, Education, Health, Voice and agency
Notes	

Risk of bias table

Bellows et al., 2011

**Table:** 

Methods	Systematic Review/Descriptive Synthesis
Participants	Low‐income pregnant women, or of childbearing age and sex workers
Interventions	Social assistance
Outcomes	Health
Notes	

Risk of bias table

Bellows et al., 2016

**Table:** 

Methods	Systematic Review/Narrative Synthesis
Participants	Women and girls
Interventions	Social assistance
Outcomes	Health
Notes	

Risk of bias table

Blacklock et al., 2016

**Table:** 

Methods	Systematic Review/Narrative Synthesis
Participants	No restrictions
Interventions	Social assistance
Outcomes	Economic security and empowerment, Health, Voice and agency
Notes	

Risk of bias table

Bourey et al., 2015

**Table:** 

Methods	Systematic Review/Qualitative Analysis
Participants	No restrictions
Interventions	Social assistance
	Labour market
Outcomes	Protection
Notes	

Risk of bias table

Brody et al., 2015

**Table:** 

Methods	Systematic Review/Meta‐analysis/Meta‐Ethnography
Participants	Women, no age restrictions
Interventions	Social insurance
	Labour market
Outcomes	Economic security and empowerment, Voice and agency, Mental and psychosocial wellbeing
Notes	

Risk of bias table

Buller et al., 2018

**Table:** 

Methods	Scoping Review/Qualitative Analysis
Participants	Low‐income households, no age restrictions
Interventions	Social assistance
Outcomes	Protection, Others
Notes	

Risk of bias table

Carroll et al., 2020

**Table:** 

Methods	Systematic Review/Qualitative Analysis
Participants	Mothers and children
Interventions	Labour market
Outcomes	Health, Others
Notes	

Risk of bias table

Chinen et al., 2017

**Table:** 

Methods	Systematic Review, Meta‐ Analysis and Narrative Meta‐Synthesis
Participants	Disadvantaged, unemployed or underemployed, 18 and above
Interventions	Social assistance, Labour market
Outcomes	Economic security and empowerment
Notes	

Risk of bias table

Choko et al., 2018

**Table:** 

Methods	Systematic Review/Meta‐and Qualitative Analysis
Participants	Men, 18 and above
Interventions	Social assistance
Outcomes	Health
Notes	

Risk of bias table

Clifford et al., 2013

**Table:** 

Methods	Systematic Review
Participants	No restrictions
Interventions	Social assistance, Labour market
Outcomes	Education
Notes	

Risk of bias table

Dammert et al., 2018

**Table:** 

Methods	Systematic Review/Descriptive synthesis
Participants	Children under age 17 in labour and hazardous labour
Interventions	Social assistance, Labour market
Outcomes	Protection
Notes	

Risk of bias table

Devereux et al., 2015

**Table:** 

Methods	Systematic Review/Qualitative Analysis
Participants	No restrictions
Interventions	Social assistance, Social insurance, Labour market
Outcomes	Other
Notes	

Risk of bias table

Dickson & Bangpan, 2012

**Table:** 

Methods	Systematic review/Meta‐analysis/thematic narrative review
Participants	Women, ages 10 to 24
Interventions	Social assistance, Labour market
Outcomes	Economic security and empowerment, Education, Health, Voice and agency, Protection, Mental and Psychosocial wellbeing, Other
Notes	

Risk of bias table

Doocy & Tappis, 2017

**Table:** 

Methods	Systematic Review/Narrative Synthesis
Participants	Population affected by humanitarian emergencies
Interventions	Social assistance, Labour market
Outcomes	Education, Health, Economic security and empowerment, Voice and agency, Protection, Mental and psychosocial wellbeing
Notes	

Risk of bias table

Dror et al., 2016

**Table:** 

Methods	Systematic Review, Meta‐analysis, Thematic analysis
Participants	No restrictions
Interventions	Social insurance
Outcomes	Health, Economic security and empowerment
Notes	

Risk of bias table

Durao et al., 2020

**Table:** 

Methods	Systematic review/Meta‐analysis
Participants	No restrictions
Interventions	Social assistance, Social insurance, Labour market
Outcomes	Economic security and empowerment, Health
Notes	

Risk of bias table

Dzakpasu et al., 2014

**Table:** 

Methods	Systematic Review/Narrative Synthesis
Participants	Women, no age restrictions
Interventions	Social assistance
Outcomes	Health
Notes	

Risk of bias table

Ensor et al., 2019

**Table:** 

Methods	Systematic Review/Thematic Synthesis
Participants	Men, ages 10 and above
Interventions	Social assistance
Outcomes	Health
Notes	

Risk of bias table

Gibbs et al., 2017

**Table:** 

Methods	Scoping Review/Qualitative Analysis
Participants	No restrictions
Interventions	Social assistance
Outcomes	Health and protection
Notes	

Risk of bias table

Glassman et al., 2013

**Table:** 

Methods	Systematic Review/Descriptive Synthesis
Participants	Mothers, New‐borns
Interventions	Social assistance
Outcomes	Health
Notes	

Risk of bias table

Haberland et al., 2018

**Table:** 

Methods	Systematic Review
Participants	Women, ages 10 to 24
Interventions	Social assistance, Labour market
Outcomes	Education, Economic security and empowerment, Health, Protection
Notes	

Risk of bias table

Halim et al., 2015

**Table:** 

Methods	Systematic Review/Qualitative Analysis
Participants	Women of reproductive age and their children
Interventions	Social care
Outcomes	Economic security and empowerment, Education, Health
Notes	

Risk of bias table

Hidrobo et al., 2018

**Table:** 

Methods	Systematic Review/Meta‐analysis
Participants	No restrictions
Interventions	Social assistance, Labour market
Outcomes	Economic security and empowerment, Health
Notes	

Risk of bias table

Hindin et al., 2016

**Table:** 

Methods	Systematic Review/Qualitative Analysis
Participants	Age 10 to 24
Interventions	Social insurance, Labour market, Social care
Outcomes	Health
Notes	

Risk of bias table

Hunter & Murray, 2017

**Table:** 

Methods	Systematic Review/Thematic Analysis
Participants	Pregnant women or women with 42 days of end of pregnancy
Interventions	Social assistance
Outcomes	Health
Notes	

Risk of bias table

Hunter et al., 2017

**Table:** 

Methods	Systematic Review/Narrative Synthesis
Participants	Pregnant women or women within 42 days of end of pregnancy
Interventions	Social assistance
Outcomes	Health
Notes	

Risk of bias table

Hurst et al., 2015

**Table:** 

Methods	Systematic Review/Narrative Synthesis
Participants	Women of Childbearing age
Interventions	Social assistance
Outcomes	Health
Notes	

Risk of bias table

Ibanez et al., 2017

**Table:** 

Methods	Systematic Review/Meta‐analysis/Narrative Synthesis
Participants	Women, no age restriction
Interventions	Labour market, Social care
Outcomes	Economic security and empowerment, Education, Health, Mental and psychosocial wellbeing, Voice and agency, Other
Notes	

Risk of bias table

Kabeer et al., 2012

**Table:** 

Methods	Systematic review/Realist Synthesis
Participants	No restrictions
Interventions	Social assistance
Outcomes	Economic security and empowerment
Notes	

Risk of bias table

Kalamar, Bayer, et al., 2016

**Table:** 

Methods	Systematic review/Qualitative analysis
Participants	Men and women, age 10 to 24
Interventions	Social assistance and labour market
Outcomes	Health
Notes	

Risk of bias table

Kalamar, Lee‐Rife, et al., 2016

**Table:** 

Methods	Systematic review/Qualitative analysis
Participants	Men and women, age 10 to 24
Interventions	Social assistance, labour market
Outcomes	Protection
Notes	

Risk of bias table

Kennedy et al., 2014

**Table:** 

Methods	Systematic review/Qualitative analysis
Participants	No restrictions
Interventions	Labour market
Outcomes	Health
Notes	

Risk of bias table

Kennedy et al., 2020

**Table:** 

Methods	Systematic review, meta‐analysis and descriptive synthesis
Participants	Men, ages 10 and above
Interventions	Social assistance
Outcomes	Health
Notes	

Risk of bias table

Khan et al., 2016

**Table:** 

Methods	Systematic review/Qualitative analysis
Participants	Women, no age restrictions
Interventions	Social assistance
Outcomes	Health
Notes	

Risk of bias table

Kluve et al., 2017

**Table:** 

Methods	Systematic review/Meta‐analysis
Participants	Men and women, age 15 to 35
Interventions	Labour market
Outcomes	Economic security and empowerment
Notes	

Risk of bias table

Kristjansson et al., 2015

**Table:** 

Methods	Systematic review/Meta‐analysis
Participants	Children age 3 months to 5 years
Interventions	Social assistance
Outcomes	Health, Mental and psychosocial wellbeing
Notes	

Risk of bias table

Kumar et al., 2018

**Table:** 

Methods	Systematic review/meta‐analysis/Narrative Synthesis
Participants	Rural and Semi urban and population
Interventions	Labour market
Outcomes	Economic security and empowerment, Other
Notes	

Risk of bias table

Langer et al., 2018

**Table:** 

Methods	Systematic review/Meta‐analysis and narrative synthesis
Participants	Women, age 15 above
Interventions	Social assistance, Social insurance, Social care and Labour market
Outcomes	Economic security and empowerment
Notes	

Risk of bias table

Lee‐Rife et al., 2012

**Table:** 

Methods	Systematic review/Descriptive Synthesis
Participants	No restrictions
Interventions	Social assistance
Outcomes	Protection
Notes	

Risk of bias table

Malhotra et al. 2021

**Table:** 

Methods	Systematic review/descriptive synthesis
Participants	Women age 0 to 24
Interventions	Social assistance, Labour market
Outcomes	Protection
Notes	

Risk of bias table

Manley and Slavchevska 2012

**Table:** 

Methods	Rapid Evidence Assessment/Meta‐analysis
Participants	No restrictions
Interventions	Social assistance
Outcomes	Health
Notes	

Risk of bias table

Maynard et al., 2017

**Table:** 

Methods	Systematic review/narrative synthesis
Participants	Population affected by humanitarian crisis
Interventions	Social assistance
Outcomes	Economic security and empowerment, Health, Mental and psychosocial wellbeing, Protection, Voice and agency, Others
Notes	

Risk of bias table

Meyer et al., 2011

**Table:** 

Methods	Systematic review/narrative synthesis
Participants	No restrictions
Interventions	Social assistance
Outcomes	Health
Notes	

Risk of bias table

Murray et al., 2014

**Table:** 

Methods	Systematic review/narrative and meta‐synthesis
Participants	Pregnant women or women within42 days of end of pregnancy
Interventions	Social assistance
Outcomes	Health, Others
Notes	

Risk of bias table

Mwaikambo et al., 2011

**Table:** 

Methods	Systematic review/Qualitative Analysis
Participants	No restrictions
Interventions	Social Assistance
Outcomes	Health, Others
Notes	

Risk of bias table

Målqvist et al., 2013

**Table:** 

Methods	Systematic review/Meta‐analysis and narrative synthesis
Participants	Mothers and children, no age restrictions
Interventions	Social assistance
Outcomes	Health
Notes	

Risk of bias table

Owusu‐Addo and Cross, 2014

**Table:** 

Methods	Systematic review/Narrative Synthesis
Participants	Mothers, caregivers, children under 19
Interventions	Social assistance
Outcomes	Health, Other
Notes	

Risk of bias table

Owusu‐Addo et al., 2018

**Table:** 

Methods	Systematic review/Narrative and thematic synthesis
Participants	No restrictions
Interventions	Social assistance
Outcomes	Economic security and empowerment, Education, Voice and agency, Health, Protection
Notes	

Risk of bias table

Oya et al., 2017

**Table:** 

Methods	Systematic review/Meta‐analysis and/Thematic Synthesis
Participants	Agricultural producers and wage workers
Interventions	Labour market
Outcomes	Economic security and empowerment, Education, Health, Voice and agency
Notes	

Risk of bias table

Pega et al., 2017

**Table:** 

Methods	Systematic review/Meta‐analysis/Narrative Synthesis
Participants	No restrictions
Interventions	Social assistance
Outcomes	Health, Education, Mental and Psychosocial wellbeing
Notes	

Risk of bias table

Pega et al., 2015

**Table:** 

Methods	Systematic review/Narrative Synthesis
Participants	Population affected by humanitarian crises
Interventions	Social assistance
Outcomes	Health
Notes	

Risk of bias table

Petrosino et al., 2012

**Table:** 

Methods	Systematic review/Meta‐analysis
Participants	Primary and secondary school students
Interventions	Social assistance
Outcomes	Education
Notes	

Risk of bias table

Santos et al., 2019

**Table:** 

Methods	Systematic review/Qualitative analysis
Participants	Children, ages 17 and under
Interventions	Social assistance
Outcomes	Education
Notes	

Risk of bias table

Skeen et. al 2017

**Table:** 

Methods	Systematic Review/Qualitative Analysis
Participants	Children affected by HIV/AIDS
Interventions	Social assistance, Labour market
Outcomes	Health
Notes	

Risk of bias table

Snilstveit et al., 2015

**Table:** 

Methods	Systematic review/Meta‐analysis/Qualitative analysis
Participants	Primary and secondary school age children
Interventions	Social assistance
Outcomes	Education
Notes	

Risk of bias table

Tirivayi et al., 2016

**Table:** 

Methods	Systematic review/Narrative synthesis
Participants	No restrictions
Interventions	Social assistance, Labour market
Outcomes	Economic security and empowerment
Notes	

Risk of bias table

Ton et al., 2013

**Table:** 

Methods	Systematic review/Qualitative analysis
Participants	Small holder farmers
Interventions	Social assistance
Outcomes	Economic security and empowerment
Notes	

Risk of bias table

Tripney et al., 2013

**Table:** 

Methods	Systematic review/Meta‐analysis
Participants	Men and women, age 15 to 24
Interventions	Labour market
Outcomes	Economic security and empowerment
Notes	

Risk of bias table

Van et al. 2019

**Table:** 

Methods	Systematic review/Narrative Synthesis
Participants	No restriction
Interventions	Social insurance
Outcomes	Economic security and empowerment, Health
Notes	

Risk of bias table

Waddington et al., 2014

**Table:** 

Methods	Systematic review/Meta‐analysis/Framework Synthesis
Participants	Farmers, no age restrictions
Interventions	Labour market
Outcomes	Economic security and empowerment, Education, Health, Other
Notes	

Risk of bias table

World Bank, 2014

**Table:** 

Methods	Systematic review/Narrative Synthesis
Participants	No restrictions
Interventions	Social assistance
Outcomes	Economic security and empowerment, Education, Health, Protection, Voice and agency
Notes	

Risk of bias table

Yoong et al., 2012

**Table:** 

Methods	Systematic review/Narrative Synthesis
Participants	No restrictions
Interventions	Social assistance, Labour market
Outcomes	Economic security and empowerment, Education, Health, Voice and agency, Mental and psychosocial wellbeing, Protection
Notes	

Risk of bias table

Zakiyah et al., 2016

**Table:** 

Methods	Systematic review/Narrative Synthesis
Participants	No restrictions
Interventions	Social assistance, Labour market
Outcomes	Economic security and empowerment, Health, Protection, Mental and psychosocial wellbeing, Education
Notes	

Risk of bias table

Zuurmond et al., 2012

**Table:** 

Methods	Systematic review/Narrative Synthesis
Participants	Men and women, age 15 to 24
Interventions	Social care
Outcomes	Health
Notes	

#### Characteristics of ongoing studies

Little et al.

**Table:** 

Study name
Methods
Participants
Interventions
Outcomes
Starting date
Contact information
Notes

## SOURCES OF SUPPORT

### Internal sources

- None, Other

No internal sources of support

### External sources

- Foreign, Commonwealth & Development Office (FCDO), UK

This systematic review has been funded by UK aid from the UK government; however the views expressed do not necessarily reflect the UK government s official policies. The systematic review is part of a research programme investigating gender‐responsive and age‐sensitive social protection systems to enhance gender equality outcomes in low and middle‐income settings. The 5‐year programme (2018–2023) is led by UNICEF Office of Research—Innocenti and funded by the UK and other partners. The systematic review is part of the first stream of the project and will inform future implementation within the programme.

## Supporting information

Supporting information.
