# Supplementary material for: Impact of social protection on gender equality in low‐ and middle‐income countries: A systematic review of reviews
Source: Campbell Syst Rev. 2022 May 25;18(2):e1240. doi: 10.1002/cl2.1240 (PMC9133545; doi:10.1002/cl2.1240)
Supplement: Supplementary file 1 — Supporting information. [file CL2-18-e1240-s001.docx]

# Appendices

1. Appendix 1 – PRISMA checklist
2. Appendix 2 - JBI Critical appraisal checklist for systematic reviews and research syntheses
3. Appendix 3 – Summary of included reviews
4. Appendix 4 – Correlation matrix of systematic reviews with overlaps

3.1 Correlation matrix of social assistance systematic reviews with high and very high overlap

3.2 Correlation matrix of labour market programmes systematic reviews with high and very high overlap

1. Appendix 5 – List of included reviews (by quality)

2.1 List of 36 high quality reviews

2.2 List of 34 moderate quality reviews

- 1. List of 15 low quality reviews (excluded from systematic review)
  2. Number of reviews that scored positively across each JBI item

1. Appendix 6 – Summary of findings from meta-analyses
2. Appendix 7 – Impact of social protection programmes on gender equality outcomes
3. Appendix 8 – Evidence gap maps

### Appendix 1 - PRISMA checklist

| **Section and Topic** | **Item #** | **Checklist item** | **Location where item is reported** |
| --- | --- | --- | --- |
| **TITLE** | | |  |
| Title | 1 | Identify the report as a systematic review. | Title |
| **ABSTRACT** | | |  |
| Abstract | 2 | See the PRISMA 2020 for Abstracts checklist. | Abstract |
| **INTRODUCTION** | | |  |
| Rationale | 3 | Describe the rationale for the review in the context of existing knowledge. | Why it is important to do this review? |
| Objectives | 4 | Provide an explicit statement of the objective(s) or question(s) the review addresses. | Objectives |
| **METHODS** | | |  |
| Eligibility criteria | 5 | Specify the inclusion and exclusion criteria for the review and how studies were grouped for the syntheses. | Criteria for considering studies for this review |
| Information sources | 6 | Specify all databases, registers, websites, organisations, reference lists and other sources searched or consulted to identify studies. Specify the date when each source was last searched or consulted. | Search methods for identification of studies |
| Search strategy | 7 | Present the full search strategies for all databases, registers and websites, including any filters and limits used. | Review protocol |
| Selection process | 8 | Specify the methods used to decide whether a study met the inclusion criteria of the review, including how many reviewers screened each record and each report retrieved, whether they worked independently, and if applicable, details of automation tools used in the process. | Selection of studies |
| Data collection process | 9 | Specify the methods used to collect data from reports, including how many reviewers collected data from each report, whether they worked independently, any processes for obtaining or confirming data from study investigators, and if applicable, details of automation tools used in the process. | Data extraction and management |
| Data items | 10a | List and define all outcomes for which data were sought. Specify whether all results that were compatible with each outcome domain in each study were sought (e.g., for all measures, time points, analyses), and if not, the methods used to decide which results to collect. | Data extraction and management |
|  | 10b | List and define all other variables for which data were sought (e.g., participant and intervention characteristics, funding sources). Describe any assumptions made about any missing or unclear information. | Review protocol |
| Study risk of bias assessment | 11 | Specify the methods used to assess risk of bias in the included studies, including details of the tool(s) used, how many reviewers assessed each study and whether they worked independently, and if applicable, details of automation tools used in the process. | Assessment of risk of bias in included reviews |
| Effect measures | 12 | Specify for each outcome the effect measure(s) (e.g. risk ratio, mean difference) used in the synthesis or presentation of results. | Measures of treatment effect |
| Synthesis methods | 13a | Describe the processes used to decide which studies were eligible for each synthesis (e.g. tabulating the study intervention characteristics and comparing against the planned groups for each synthesis (item #5)). | Data synthesis |
|  | 13b | Describe any methods required to prepare the data for presentation or synthesis, such as handling of missing summary statistics, or data conversions. | Unit of analysis |
|  | 13c | Describe any methods used to tabulate or visually display results of individual studies and syntheses. | Data synthesis |
|  | 13d | Describe any methods used to synthesize results and provide a rationale for the choice(s). If meta-analysis was performed, describe the model(s), method(s) to identify the presence and extent of statistical heterogeneity, and software package(s) used. | Data synthesis |
|  | 13e | Describe any methods used to explore possible causes of heterogeneity among study results (e.g., subgroup analysis, meta-regression). | Subgroup analysis and investigation of heterogeneity |
|  | 13f | Describe any sensitivity analyses conducted to assess robustness of the synthesized results. | Not applicable |
| Reporting bias assessment | 14 | Describe any methods used to assess risk of bias due to missing results in a synthesis (arising from reporting biases). | Assessment of risk of bias in included reviews |
| Certainty assessment | 15 | Describe any methods used to assess certainty (or confidence) in the body of evidence for an outcome. | Assessment of risk of bias in included reviews |
| **RESULTS** | | |  |
| Study selection | 16a | Describe the results of the search and selection process, from the number of records identified in the search to the number of studies included in the review, ideally using a flow diagram. | Results of the search |
|  | 16b | Cite studies that might appear to meet the inclusion criteria, but which were excluded, and explain why they were excluded. | Results of the search |
| Study characteristics | 17 | Cite each included study and present its characteristics. | Included studies |
| Risk of bias in studies | 18 | Present assessments of risk of bias for each included study. | Risk of bias in included reviews |
| Results of individual studies | 19 | For all outcomes, present, for each study: (a) summary statistics for each group (where appropriate) and (b) an effect estimate and its precision (e.g. confidence/credible interval), ideally using structured tables or plots. | Supporting information |
| Results of syntheses | 20a | For each synthesis, briefly summarise the characteristics and risk of bias among contributing studies. | Not applicable - low quality reviews excluded |
|  | 20b | Present results of all statistical syntheses conducted. If meta-analysis was done, present for each the summary estimate and its precision (e.g. confidence/credible interval) and measures of statistical heterogeneity. If comparing groups, describe the direction of the effect. | Supporting information |
|  | 20c | Present results of all investigations of possible causes of heterogeneity among study results. | Not applicable |
|  | 20d | Present results of all sensitivity analyses conducted to assess the robustness of the synthesized results. | Not applicable |
| Reporting biases | 21 | Present assessments of risk of bias due to missing results (arising from reporting biases) for each synthesis assessed. | Limitations and potential biases in the review process |
| Certainty of evidence | 22 | Present assessments of certainty (or confidence) in the body of evidence for each outcome assessed. | Assessment of risk of bias in included reviews |
| **DISCUSSION** | | |  |
| Discussion | 23a | Provide a general interpretation of the results in the context of other evidence. | Discussion |
|  | 23b | Discuss any limitations of the evidence included in the review. | Overall completeness and applicability of evidence |
|  | 23c | Discuss any limitations of the review processes used. | Limitations and potential biases in the review process |
|  | 23d | Discuss implications of the results for practice, policy, and future research. | Implications for practice and policy and Implications for research |
| **OTHER INFORMATION** | | |  |
| Registration and protocol | 24a | Provide registration information for the review, including register name and registration number, or state that the review was not registered. | Linked in publication heading |
|  | 24b | Indicate where the review protocol can be accessed, or state that a protocol was not prepared. | Linked in publication heading |
|  | 24c | Describe and explain any amendments to information provided at registration or in the protocol. | Differences between protocol and review |
| Support | 25 | Describe sources of financial or non-financial support for the review, and the role of the funders or sponsors in the review. | Sources of support |
| Competing interests | 26 | Declare any competing interests of review authors. | Declarations of interest |
| Availability of data, code and other materials | 27 | Report which of the following are publicly available and where they can be found: template data collection forms; data extracted from included studies; data used for all analyses; analytic code; any other materials used in the review. | Review protocol |

### Appendix 2 – JBI Critical appraisal checklist for systematic reviews and research syntheses

| 1. Is the review question clearly and explicitly stated? | Yes | No | Unclear | Not Applicable |
| --- | --- | --- | --- | --- |
| *The review question is an essential step in the systematic review process. A well-articulated question defines the scope of the review and aids in the development of the search strategy to locate the relevant evidence. An explicitly stated question, formulated around its PICO (Population, Intervention, Comparator, Outcome) elements aids both the review team in the conduct of the review and the reader in determining if the review has achieved its objectives. Ideally the review question should be articulated in a published protocol; however this will not always be the case with many reviews that are located.* | | | | |
| 1. Were the inclusion criteria appropriate for the review question? | Yes | No | Unclear | Not Applicable |
| *The inclusion criteria should be identifiable from, and match the review question. The necessary elements of the PICO should be explicit and clearly defined. The inclusion criteria should be detailed and the included reviews should clearly be eligible when matched against the stated inclusion criteria. Appraisers of meta-analyses will find that inclusion criteria may include criteria around the ability to conduct statistical analyses which would not be the norm for a systematic review. The types of included studies should be relevant to the review question, for example, an umbrella review aiming to summarize a range of effective non-pharmacological interventions for aggressive behaviors amongst elderly patients with dementia will limit itself to including systematic reviews and meta-analyses that synthesize quantitative studies assessing the various interventions; qualitative or economic reviews would not be included.* | | | | |
| 1. Was the search strategy appropriate? | Yes | No | Unclear | Not Applicable |
| *A systematic review should provide evidence of the search strategy that has been used to locate the evidence. This may be found in the methods section of the review report in some cases, or as an appendix that may be provided as supplementary information to the review publication. A systematic review should present a clear search strategy that addresses each of the identifiable PICO components of the review question. Some reviews may also provide a description of the approach to searching and how the terms that were ultimately used were derived, though due to limits on word counts in journals this may be more the norm in online only publications. There should be evidence of logical and relevant keywords and terms and also evidence that Subject Headings and Indexing terms have been used in the conduct of the search. Limits on the search should also be considered and their potential impact; for example, if a date limit was used, was this appropriate and/or justified? If only English language studies were included, will such a language bias have an impact on the review? The response to these considerations will depend, in part, on the review question.* | | | | |
| 1. Were the sources and resources used to search for studies adequate? | Yes | No | Unclear | Not Applicable |
| *A systematic review should attempt to identify “all” the available evidence and as such there should be evidence of a comprehensive search strategy. Multiple electronic databases should be searched including major bibliographic citation databases such as MEDLINE and CINAHL. Ideally, other databases that are relevant to the review question should also be searched, for example, a systematic review with a question about a physical therapy intervention should also look to search the PEDro database, whilst a review focusing on an educational intervention should also search the ERIC. Reviews of effectiveness should aim to search trial registries. A comprehensive search is the ideal way to minimize publication bias, as a result, a well conducted systematic review should also attempt to search for grey literature, or “unpublished” studies; this may involve searching websites relevant to the review question, or thesis repositories.* | | | | |
| 1. Were the criteria for appraising studies appropriate? | Yes | No | Unclear | Not Applicable |
| *The systematic review should present a clear statement that critical appraisal was conducted and provide the details of the items that were used to assess the included studies. This may be presented in the methods of the review, as an appendix of supplementary information, or as a reference to a source that can be located. The tools or instruments used should be appropriate for the review question asked and the type of research conducted. For example, a systematic review of effectiveness should present a tool or instrument that addresses aspects of validity for experimental studies and randomized controlled trials such as randomization and blinding – if the review includes observational research to answer the same question a different tool would be more appropriate. Similarly, a review assessing diagnostic test accuracy may refer to the recognized QUADAS1 tool.* | | | | |
| 1. Was critical appraisal conducted by two or more reviewers independently? | Yes | No | Unclear | Not Applicable |
| *Critical appraisal or some similar assessment of the quality of the literature included in a systematic review is essential. A key characteristic to minimize bias or systematic error in the conduct of a systematic review is to have the critical appraisal of the included studies completed independently and in duplicate by members of the review team. The systematic review should present a clear statement that critical appraisal was conducted by at least two reviewers working independently from each other and conferring where necessary to reach decision regarding study quality and eligibility on the basis of quality.* | | | | |
| 1. Were there methods to minimise errors in data extraction? | Yes | No | Unclear | Not Applicable |
| Efforts made by review authors during data extraction can also minimize bias or systematic errors in the conduct of a *systematic* review. Strategies to minimize bias may include conducting all data extraction in duplicate and independently, using specific tools or instruments to guide data extraction and some evidence of piloting or training around their use. | | | | |
| 1. Were the methods used to combine studies appropriate? | Yes | No | Unclear | Not Applicable |
| *A synthesis of the evidence is a key feature of a systematic review. The synthesis that is presented should be appropriate for the review question and the stated type of systematic review and evidence it refers to. If a meta-analysis* has *been conducted this needs to be reviewed carefully. Was it appropriate to combine the studies? Have the reviewers assessed heterogeneity statistically and provided some explanation for heterogeneity that may be present? Often, where heterogeneous studies are included in the systematic review, narrative synthesis will be an appropriate method for presenting the results of multiple studies. If a qualitative review, are the methods that have been used to synthesize findings congruent with the stated methodology of the review? Is there adequate descriptive and explanatory information to support the final synthesized findings that have been constructed from the findings sourced from the original research?* | | | | |
| 1. Was the likelihood of publication bias assessed? | Yes | No | Unclear | Not Applicable |
| *As mentioned, a comprehensive search strategy is the best means by which a review author may alleviate the impact of publication bias on the results of the review. Reviews may also present statistical tests such as Egger’s test or funnel plots to also assess the potential presence of publication bias and its potential impact on the results of the review. This question will not be applicable to systematic reviews of qualitative evidence.* | | | | |
| 1. Were recommendations for policy and/or practice supported by the reported data? | Yes | No | Unclear | Not Applicable |
| *Whilst the first nine (9) questions specifically look to identify potential bias in the conduct of a systematic review, the final questions are more indictors of review quality rather than validity. Ideally a review should present recommendations for policy and practice. Where these recommendations are made there should be a clear link to the results of the review. Is there evidence that the strength of the findings and the quality of the research been considered in the formulation of review recommendations?* | | | | |
| 1. Were the specific directives for new research appropriate? | Yes | No | Unclear | Not Applicable |
| *The systematic review process is recognized for its ability to identify where gaps in the research, or knowledge base, around a particular topic exist. Most systematic review authors will provide some indication, often in the discussion section of the report, of where future research direction should lie. Where evidence is scarce or sample sizes that support overall estimates of effect are small and effect estimates are imprecise, repeating similar research to those identified by the review may be necessary and appropriate. In other instances, the case for new research questions to investigate the topic may be warranted.* | | | | |
| **Overall appraisal:**  Include □  Exclude □  Seek further info □  **Comments (Including reason for exclusion):** | | | | |

*Aromataris et al.2015*

### Appendix 3 – Summary of included reviews

| **Authors (Year)** | **No. of included studies** | **Type of Review / Analysis** | **Geographic Focus** | **Gender and Life Course** | **Intervention Category** | **Outcome Area** |
| --- | --- | --- | --- | --- | --- | --- |
| Adebayo et al. (2015) | 25 | Systematic Review / Narrative synthesis | LMICs | No restrictions | Social insurance | 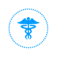 |
| Aitken et al. (2015) | 7 | Systematic Review | Global | Working age women | Labour market | 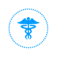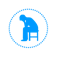 |
| Baird et al. (2013) | 75 | Systematic Review / Meta-analysis | LMICs | Low-income households with school-aged children | Social assistance | 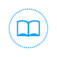 |
| Banks et al. (2017) | 15 | Systematic Review / Qualitative analysis | LMICs | Persons with disabilities, no age restrictions | Social assistance Social insurance | Other |
| Bassani et al. (2013) | 25 | Systematic Review / Qualitative analysis | LMICs | Children under age 6 | Social assistance | 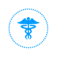 |
| Bastagli et al. (2016) | 201 | Systematic Review / Narrative synthesis | LMICs | No restrictions | Social assistance | 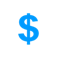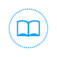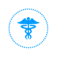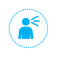 |
| Bellows et al. (2011) | 13 | Systematic Review / Descriptive synthesis | LMICs | Low-income pregnant women, or of child-bearing age and sex workers | Social assistance | 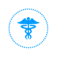 |
| Bellows et al. (2016) | 16 | Systematic Review / Narrative synthesis | LMICs | Women and girls | Social assistance | 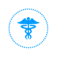 |
| Blacklock et al. (2016) | 13 | Systematic Review / Narrative synthesis | LMICs | No restrictions | Social assistance | 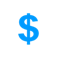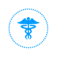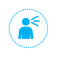 |
| Bourey et al. (2015) | 20 | Systematic Review / Qualitative analysis | LMICs | No restrictions | Social assistance Labour market | 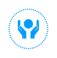 |
| Brody et al. (2015) | 34 | Systematic Review / Meta-analysis and meta-ethnography | LMICs | Women, no age restrictions | Social insurance Labour market | 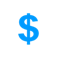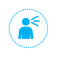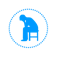 |
| Buller et al. (2018) | 22 | Scoping Review / Qualitative analysis | LMICs | Low-income households, no age restrictions | Social assistance | 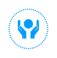 Other |
| Carroll et al. (2020) | 45 | Systematic Review / Qualitative analysis | Global | Mothers and children | Labour market | 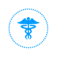 Other |
| Chinen et al. (2017) | 35 | Systematic Review / Meta-analysis and narrative meta-synthesis | LMICs | Disadvantaged, unemployed or underemployed women, 18 and above | Social assistance Labour market | 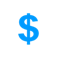 |
| Choko et al. (2018) | 9 | Systematic Review / Meta-analysis and qualitative analysis | LMICs | Men, 18 and above | Social assistance | 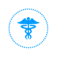 |
| Clifford et al. (2013) | 175 | Systematic Review | LMICs | No restrictions | Social assistance Labour market | 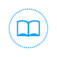 |
| Dammert et al. (2018) | 33 | Systematic Review / Descriptive synthesis | LMICs | Children under age 17 in labour and hazardous labour | Social assistance Labour market | 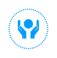 |
| Devereux et al. (2015) | 85 | Systematic Review / Qualitative analysis | LMICs | No restrictions | Social assistance Social insurance Labour market | Other |
| Dickson & Bangpan (2012) | 29 | Systematic Review / Meta-analysis and thematic narrative synthesis | LMICs | Women, ages 10 to 24 | Social assistance Labour market | 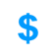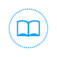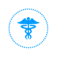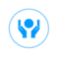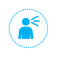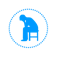 Other |
| Doocy et al. (2017) | 113 | Systematic Review / Narrative synthesis | Global / Humanitarian emergencies | Populations affected by humanitarian emergencies | Social assistance Labour market | 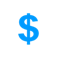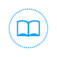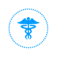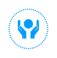 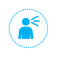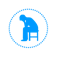 |
| Dror et al. (2016) | 54 | Systematic Review / Meta-analysis and thematic synthesis | LMICs | No restrictions | Social insurance | 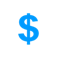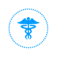 |
| Durao et al. (2020) | 59 | Systematic Review / Meta-analysis | LMICs | No restrictions | Social assistance Social insurance Labour market | 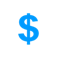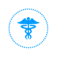 |
| Dzakpasu et al. (2014) | 20 | Systematic Review / Narrative synthesis | LMICs | Women, no age restrictions | Social assistance | 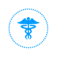 |
| Ensor et al. (2019) | 18 | Systematic Review / Thematic synthesis | LMICs | Men, ages 10 and above | Social assistance | 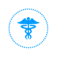 |
| Gibbs et al. (2017) | 45 | Scoping Review / Qualitative analysis | Global | No restrictions | Social assistance | 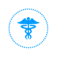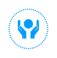 |
| Glassman et al. (2013) | 24 | Systematic Review / Descriptive synthesis | LMICs | Mothers and new-borns | Social assistance | 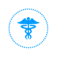 |
| Haberland et al. (2018) | 19 | Systematic Review | LMICs | Women, ages 10 to 24 | Social assistance Labour market | 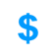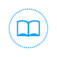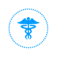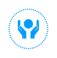 |
| Halim et al. (2015) | 29 | Systematic Review / Qualitative analysis | LMICs | Women of reproductive age and their children | Social care | 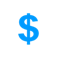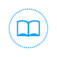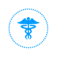 |
| Hidrobo et al. (2018) | 9 | Systematic Review / Meta-analysis | LMICs | No restrictions | Social assistance Labour market | 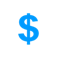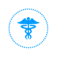 |
| Hindin et al. (2016) | 17 | Systematic Review / Qualitative analysis | LMICs | Ages 10 to 24 | Social assistance Labour market Social care | 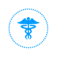 |
| Hunter & Murray (2017) | 49 | Systematic Review / Thematic synthesis | LMICs | Pregnant women or women within 42 days of end of pregnancy | Social assistance | 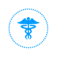 |
| Hunter et al. (2017) | 51 | Systematic Review / Narrative synthesis | LMICs | Pregnant women or women within 42 days of end of pregnancy | Social assistance | 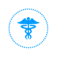 |
| Hurst et al. (2015) | 16 | Systematic Review / Narrative synthesis | LMICs | Women of childbearing age | Social assistance | 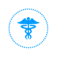 |
| Ibanez et al. (2017) | 35 | Systematic Review / Meta-analysis and narrative synthesis | LMICs | Women, no age restrictions | Labour market Social care | 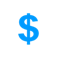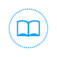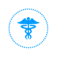 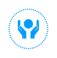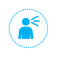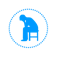 Other |
| Kabeer et al. (2012) | 46 | Systematic Review / Realist synthesis | Global | No restrictions | Social assistance | 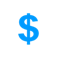 |
| Kalamar, Bayer et al. (2016) | 21 | Systematic Review / Qualitative analysis | LMICs | Men and women, ages 10 to 24 | Social assistance Labour market | 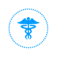 |
| Kalamar, Lee-Rife et al. (2016) | 11 | Systematic Review / Qualitative analysis | LMICs | Men and women, ages 10 to 24 | Social assistance Labour market | 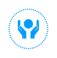 |
| Kennedy et al. (2014) | 12 | Systematic Review / Qualitative analysis | LMICs | No restrictions | Labour market | 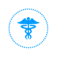 |
| Kennedy et al. (2020) | 8 | Systematic Review / Meta-analysis and descriptive synthesis | LMICs | Men, ages 10 and above | Social assistance | 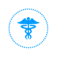 |
| Khan et al. (2016) | 11 | Systematic Review / Qualitative analysis | LMICs | Women, no age restrictions | Social assistance | 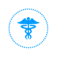 |
| Kluve et al. (2017) | 113 | Systematic Review / Meta-analysis | Global | Men and women, ages 15 to 35 | Labour market | 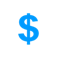 |
| Kristjansson et al. (2015) | 32 | Systematic Review / Meta-analysis | Global | Children age 3 months to 5 years | Social assistance | 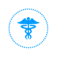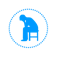 |
| Kumar et al. (2018) | 37 | Systematic Review / Meta-analysis and narrative synthesis | LMICs | Rural and semi-urban populations | Labour market | 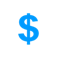 Other |
| Langer et al. (2018) | 19 | Systematic Review / Meta-analysis and narrative synthesis | LMICs | Women, ages 15 and above | Social assistance Social insurance Labour market Social care | 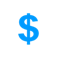 |
| Lee-Rife et al. (2012) | 34 | Systematic Review / Descriptive synthesis | Low-income countries | No restrictions | Social assistance | 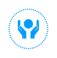 |
| Malhotra & Elnakib (2021) | 34 | Systematic Review / Descriptive synthesis | LMICs | Women, age 0 to 24 | Social assistance Labour market | 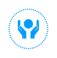 |
| Målqvist et al. (2013) | 18 | Systematic Review / Meta-analysis and narrative synthesis | LMICs | Mothers and children, no age restrictions | Social assistance | 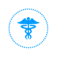 |
| Manley et al. (2012) | 24 | Rapid Evidence Assessment / Meta-analysis | LMICs | No restrictions | Social assistance | 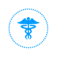 |
| Maynard et al. (2017) | 11 | Systematic Review / Narrative synthesis | LMICs | Populations affected by humanitarian crises | Social assistance | 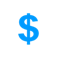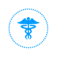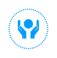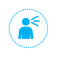 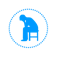  Other |
| Meyer et al. (2011) | 24 | Systematic Review / Narrative synthesis | LMICs | No restrictions | Social assistance | 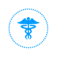 |
| Murray et al. (2014) | 72 | Systematic Review / Narrative and meta-synthesis | LMICs | Pregnant women or women within 42 days of end of pregnancy | Social assistance | 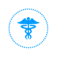 Other |
| Mwaikambo et al. (2011) | 63 | Systematic Review / Qualitative analysis | LMICs | No restrictions | Social assistance | 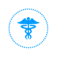 Other |
| Owusu-Addo & Cross (2014) | 16 | Systematic Review / Narrative synthesis | LMICs | Mothers, caregivers, and children under 19 | Social assistance | 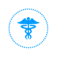 Other |
| Owusu-Addo et al. (2018) | 53 | Systematic Review / Narrative and thematic synthesis | LMICs | No restrictions | Social assistance | 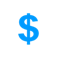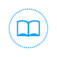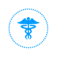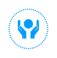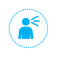 Other |
| Oya et al. (2017) | 179 | Systematic Review / Meta-analysis and thematic synthesis | LMICs | Agricultural producers and wage workers | Labour market | 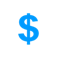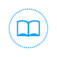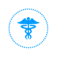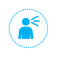 |
| Pega et al. (2015) | 3 | Systematic Review / Narrative synthesis | LMICs | Populations affected by humanitarian crises | Social assistance | 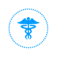 |
| Pega et al. (2017) | 21 | Systematic Review / Meta-analysis and narrative synthesis | LMICs | No restrictions | Social assistance | 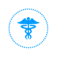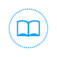 |
| Petrosino et al. (2012) | 73 | Systematic Review / Meta-analysis | LMICs | Primary and secondary school students | Social assistance |  |
| Santos et al. (2019) | 12 | Systematic Review / Qualitative analysis | Middle-income countries | Children, ages 17 and under | Social assistance |  |
| Skeen et al. (2017) | 19 | Systematic Review / Qualitative analysis | Global | Children affected by HIV/AIDS | Social assistance Labour market |  |
| Snilstveit et al. (2016) | 420 | Systematic Review / Meta-analysis and qualitative analysis | LMICs | Primary and secondary school age children | Social assistance |  |
| Tirivayi et al. (2016) | 12 | Systematic Review / Narrative synthesis | LMICs | No restrictions | Social assistance Labour market |  |
| Ton et al. (2013) | 62 | Systematic Review / Qualitative analysis | LMICs | Smallholder farmers | Social assistance |  |
| Tripney et al. (2013) | 26 | Systematic Review / Meta-analysis | LMICs | Men and women, ages 15 to 24 | Labour market |  |
| van Hees et al. (2019) | 44 | Systematic Review / Narrative synthesis | LMICs | No restrictions | Social insurance |  |
| Waddington et al. (2014) | 134 | Systematic Review / Meta-analysis and framework synthesis | LMICs | Farmers, no age restrictions | Labour market | Other |
| World Bank (2014) | 145 | Systematic Review / Narrative synthesis | LMICs | No restrictions | Social assistance |  |
| Yoong et al. (2012) | 15 | Systematic Review / Narrative synthesis | Global | No restrictions | Social assistance Labour market |  |
| Zakiyah et al. (2016) | 9 | Systematic Review / Narrative synthesis | LMICs | Women of child-bearing age | Social care | Other |
| Zuurmond et al. (2012) | 21 | Systematic Review / Narrative synthesis | LMICs | Men and women, ages 15 to 24 | Social care |  |

Health; Economic security and empowerment; Education; Safety and Protection; Mental health and Psychosocial Wellbeing; Voice and Agency

### Appendix 4 - Correlation matrix of systematic reviews with overlaps

Note: Some reviews in these tables share a slight overlap, empty spaces do not always equal no overlap.

#### 3.1 Correlation matrix of social assistance systematic reviews with high and very high overlap

|  | Bellows et al. (2016) | Ensor et al. (2019) | Gibbs et al. (2017) | Hunter & Murray (2017) | Hunter et al.(2017) | Kalamar, Lee-Rife, et al. (2016) | Kalamar, Bayer, et al. (2016) | Kennedy et al. (2020) | Khan et al. (2016) | Langer et al. (2018) | Malhotra & Elnakib (2021) | Meyer et al. (2011) | Murray et al. (2014) | Owusu-Addo & Cross (2014) | World Bank (2014) | Bassani et al. (2013) | Malqvist et al. (2013) |
| --- | --- | --- | --- | --- | --- | --- | --- | --- | --- | --- | --- | --- | --- | --- | --- | --- | --- |
| Baird et al. (2013) |  |  |  |  |  |  |  |  |  |  |  |  |  |  | 10.33% |  |  |
| Bellows et al. (2011) | 10.00% |  |  |  |  |  |  |  |  |  |  | 26.83% |  |  |  |  |  |
| Bourey et al. (2015) |  |  | 12.07% |  |  |  |  |  |  |  |  |  |  |  |  |  |  |
| Chinen et al. (2017) |  |  |  |  |  |  |  |  |  | 12.00% |  |  |  |  |  |  |  |
| Choko et al. (2018) |  | 12.50% |  |  |  |  |  | 16.67% |  |  |  |  |  |  |  |  |  |
| Ensor et al. (2019) |  |  |  |  |  |  |  | 57.89% |  |  |  |  |  |  |  |  |  |
| Glassman et al. (2013) |  |  |  | 10.00% | 13.43% |  |  |  |  |  |  |  |  |  |  |  |  |
| Hindin et al. (2016) |  |  |  |  |  | 18.52% | 23.53% |  | 10.34% |  |  |  |  |  |  |  |  |
| Hunter & Murray (2017) |  |  |  |  | 45.00% |  |  |  |  |  |  |  | 37.50% |  |  |  |  |
| Hunter et al.(2017) |  |  |  |  |  |  |  |  |  |  |  |  | 20.00% |  |  |  | 13.33% |
| Kalamar, Lee-Rife, et al. (2016) |  |  |  |  |  |  |  |  | 10.00% |  | 21.05% |  |  |  |  |  |  |
| Lee-Rife et al. (2012) |  |  |  |  |  |  |  |  |  |  | 13.11% |  |  |  |  |  |  |
| Manley et al. (2012) |  |  |  |  |  |  |  |  |  |  |  |  |  | 16.67% |  |  |  |
| Owusu-Addo & Cross (2014) |  |  |  |  |  |  |  |  |  |  |  |  |  |  |  | 13.89% |  |

#### 3.2 Correlation matrix of labour market programmes systematic reviews with high and very high overlap

|  | Kalamar, Lee-Rife, et al. (2016) | Kalamar, Bayer, et al. (2016) | Langer et al. (2018) | Malhotra & Elnakib (2021) | Yoong et al. (2012) |
| --- | --- | --- | --- | --- | --- |
| Chinen et al. (2017) |  |  | 12.00% |  |  |
| Hindin et al. (2016) | 18.52% | 23.53% |  |  |  |
| Ibanez et al. (2017) |  |  |  |  | 10.87% |
| Kalamar, Lee-Rife, et al. (2016) |  |  |  | 21.05% |  |

### Appendix 5 – List of included reviews

#### 2.1 List of 36 high-confidence reviews

| Authors | Year | Confidence | Title |
| --- | --- | --- | --- |
| Aitken et al. | 2015 | High | The maternal health outcomes of paid maternity leave: A systematic review |
| Baird et al. | 2013 | High | Relative effectiveness of conditional and unconditional cash transfers for schooling outcomes in developing countries: A systematic review |
| Bastagli et al. | 2016 | High | Cash transfers: what does the evidence say? A rigorous review of programme impact and of the role of design and implementation features |
| Blacklock et al. | 2016 | High | Paying for performance to improve the delivery and uptake of family planning in low and middle income countries: A systematic review |
| Brody et al. | 2015 | High | Economic self‐help group programs for improving women's empowerment: A systematic review |
| Chinen et al. | 2017 | High | Vocational and business training to improve women's labour market outcomes in low‐ and middle‐income countries: A systematic review |
| Clifford et al. | 2013 | High | How effective are different approaches to higher education provision in increasing access, quality and completion for students in developing countries? Does this differ by gender of students? A systematic review |
| Dickson and Bangpan | 2012 | High | Providing access to economic assets for girls and young women in low-and-lower middle-income countries: A systematic review of the evidence |
| Doocy et al. | 2017 | High | Cash‐based approaches in humanitarian emergencies: A systematic review |
| Dror et al. | 2016 | High | What factors affect voluntary uptake of community-based health insurance schemes in low- and middle-income countries? A systematic review and meta-analysis |
| Durao et al. | 2020 | High | Community-level interventions for improving access to food in low- and middle-income countries |
| Haberland et al. | 2018 | High | A systematic review of adolescent girl program implementation in low- and middle-income countries: Evidence gaps and insights |
| Hindin et al. | 2016 | High | Interventions to prevent unintended and repeat pregnancy among young people in low- and middle-income countries: A systematic review of the published and gray literature |
| Hunter et al. | 2017 | High | The effects of cash transfers and vouchers on the use and quality of maternity care services: A systematic review |
| Hunter and Murray | 2017 | High | Demand-side financing for maternal and newborn health: what do we know about factors that affect implementation of cash transfers and voucher programmes? |
| Ibanez et al. | 2017 | High | Women's economic empowerment at the community level has a positive impact on human development in low and middle income countries |
| Kluve et al. | 2017 | High | Interventions to improve the labour market outcomes of youth: A systematic review |
| Kristjansson et al. | 2015 | High | Food supplementation for improving the physical and psychosocial health of socio-economically disadvantaged children aged three months to five years: A systematic review |
| Kumar et al. | 2018 | High | The effectiveness of market led development approaches: A systematic review |
| Langer et al. | 2018 | High | Women in wage labour: A systematic review of the effectiveness and design features of interventions supporting women's participation in wage labour in higher growth and/or male-dominated sectors in low- and middle-income countries |
| Malhotra et al. | 2021 | High | 20 years of the evidence base on what works to prevent child marriage: a systematic review |
| Målqvist et al. | 2013 | High | Targeted interventions for improved equity in maternal and child health in low- and middle-income settings: A systematic review and meta-analysis |
| Manley et al. | 2012 | High | How effective are cash transfer programmes at improving nutritional status? A rapid evidence assessment of programmes’ effects on anthropometric outcomes |
| Maynard et al. | 2017 | High | The effectiveness and efficiency of interventions supporting shelter self-recovery following humanitarian crises |
| Meyer et al. | 2011 | High | The impact of vouchers on the use and quality of health goods and services in developing countries: a systematic review |
| Mwaikambo et al. | 2011 | High | What works in family planning interventions: A systematic review of the evidence |
| Owusu-Addo et al. | 2018 | High | The impact of cash transfers on social determinants of health and health inequalities in sub-Saharan Africa: A systematic review |
| Oya et al. | 2017 | High | Effects of certification schemes for agricultural production on socio-economic outcomes in low- and middle-income countries: A systematic review |
| Pega et al. | 2015 | High | Unconditional cash transfers for assistance in humanitarian disasters: Effect on use of health services and health outcomes in low- and middle-income countries |
| Pega et al. | 2017 | High | Unconditional cash transfers for reducing poverty and vulnerabilities: Effect on use of health services and health outcomes in low‐ and middle‐income countries |
| Petrosino et al. | 2012 | High | Interventions in developing nations for improving primary and secondary school enrollment of children: A systematic review |
| Snilstveit et al. | 2016 | High | Interventions for improving learning outcomes and access to education in low- and middle- income countries. A systematic review |
| Tripney et al. | 2013 | High | Technical and vocational education and training (TVET) interventions to improve the employability and employment of young people in low- and middle-income countries: A systematic review |
| van Hees et al. | 2019 | High | Leaving no one behind? Social inclusion of health insurance in low- and middle-income countries: A systematic review |
| Waddington et al. | 2014 | High | Farmer field schools for improving farming practices and farmer outcomes: A systematic review |
| Yoong et al. | 2012 | High | The impact of economic resource transfers to women versus men: a systematic review |

#### 2.2 List of 34 medium-confidence reviews

| Authors | Year | Confidence | Title |
| --- | --- | --- | --- |
| Adebayo et al. | 2015 | Moderate | A systematic review of factors that affect uptake of community-based health insurance in low-income and middle-income countries |
| Banks et al. | 2017 | Moderate | Disability and social protection programmes in low- and middle-income countries: A systematic review |
| Bassani et al. | 2013 | Moderate | Financial incentives and coverage of child health interventions: A systematic review and meta-analysis |
| Bellows et al. | 2011 | Moderate | The use of vouchers for reproductive health services in developing countries: Systematic review |
| Bellows et al. | 2016 | Moderate | Family planning vouchers in low and middle income countries: A systematic review |
| Bourey et al. | 2015 | Moderate | Systematic review of structural interventions for intimate partner violence in low- and middle-income countries: Organizing evidence for prevention |
| Buller et al. | 2018 | Moderate | A mixed-method review of cash transfers and intimate partner violence in low- and middle-income countries |
| Carroll et al. | 2020 | Moderate | A systematic review of costing studies for implementing and scaling-up breastfeeding interventions: What do we know and what are the gaps? |
| Choko et al. | 2018 | Moderate | The effect of demand-side financial incentives for increasing linkage into HIV treatment and voluntary medical male circumcision: A systematic review and meta-analysis of randomised controlled trials in low- and middle-income countries |
| Dammert et al. | 2018 | Moderate | Effects of public policy on child labor: Current knowledge, gaps, and implications for program design |
| Devereux et al. | 2015 | Moderate | Evaluating the targeting effectiveness of social transfers: A literature review |
| Dzakpasu et al. | 2014 | Moderate | Impact of user fees on maternal health service utilization and related health outcomes: A systematic review |
| Ensor et al. | 2019 | Moderate | The effectiveness of demand creation interventions for voluntary male medical circumcision for HIV prevention in sub-Saharan Africa: A mixed methods systematic review |
| Gibbs et al. | 2017 | Moderate | A global comprehensive review of economic interventions to prevent intimate partner violence and HIV risk behaviours |
| Glassman et al. | 2013 | Moderate | Impact of conditional cash transfers on maternal and newborn health |
| Halim et al. | 2015 | Moderate | The economic consequences of selected maternal and early childhood nutrition interventions in low- and middle-income countries: a review of the literature, 2000-2013. |
| Hidrobo et al. | 2018 | Moderate | Social protection, food security, and asset formation |
| Hurst et al. | 2015 | Moderate | Demand-side interventions for maternal care: Evidence of more use, not better outcomes |
| Kabeer et al. | 2012 | Moderate | What are the economic impacts of conditional cash transfer programmes? A systematic review of the evidence |
| Kalamar, Lee-Rife et al. | 2016 | Moderate | Interventions to prevent child marriage among young people in low- and middle-income countries: A systematic review of the published and gray literature |
| Kalamar, Bayer et al. | 2016 | Moderate | Interventions to prevent sexually transmitted infections, including HIV, among young people in low- and middle-income countries: A systematic review of the published and gray literature |
| Kennedy et al. | 2020 | Moderate | Economic compensation interventions to increase uptake of voluntary medical male circumcision for HIV prevention: A systematic review and meta-analysis |
| Kennedy et al. | 2014 | Moderate | A systematic review of income generation interventions, including microfinance and vocational skills training, for HIV prevention |
| Khan et al. | 2016 | Moderate | Conditional and unconditional cash transfers to improve use of contraception in low and middle income countries: A systematic review |
| Lee-Rife et al. | 2012 | Moderate | What works to prevent child marriage: A review of the evidence |
| Murray et al. | 2014 | Moderate | Effects of demand-side financing on utilisation, experiences and outcomes of maternity care in low- and middle-income countries: A systematic review |
| Owusu-Addo et al. | 2014 | Moderate | The impact of conditional cash transfers on child health in low- and middle-income countries: A systematic review |
| Santos et al. | 2019 | Moderate | The Bolsa Família Program and educational indicators of children, adolescents, and schools in Brazil: A systematic review |
| Skeen et al. | 2017 | Moderate | Interventions to improve psychosocial well-being for children affected by HIV and AIDS: A systematic review |
| Tirivayi et al. | 2016 | Moderate | The interaction between social protection and agriculture: A review of evidence |
| Ton et al. | 2013 | Moderate | Effectiveness of innovation grants to smallholder agricultural producers: An explorative systematic review |
| World Bank | 2014 | Moderate | Social safety nets and gender: Learning from impact evaluations and World Bank projects |
| Zakiyah et al. | 2016 | Moderate | Economic evaluation of family planning interventions in low and middle income countries; A systematic review |
| Zuurmond et al. | 2012 | Moderate | The effectiveness of youth centers in increasing use of sexual and reproductive health services: a systematic review |

#### 2.3 List of 15 low-confidence reviews (excluded from review)

| Authors | Year | Confidence | Title |
| --- | --- | --- | --- |
| Berti et al. | 2018 | Low | Multiple‐micronutrient supplementation: Evidence from large‐scale prenatal programmes on coverage, compliance and impact |
| Buillon and Tejerina | 2006 | Low | Do we know what works? A systematic review of impact evaluations of social programs in Latin America and the Caribbean |
| Carrasco et al. | 2018 | Low | Systematic review of the effect of economic compensation and incentives on uptake of voluntary medical male circumcision among men in sub-Saharan Africa |
| Cho and Honorati | 2014 | Low | Entrepreneurship programs in developing countries: A meta regression analysis |
| Cooper et al. | 2020 | Low | Cash transfer programs have differential effects on health: A review of the literature from low and middle-income countries |
| de Hoop and Rosati | 2014 | Low | Cash transfers and child labor |
| de Souza Cruz et al. | 2017 | Low | Conditional cash transfers and the creation of equal opportunities of health for children in low and middle-income countries: A literature review |
| Evans and Popova | 2014 | Low | Cash transfers and temptation goods: A review of global evidence |
| Fernald et al. | 2012 | Low | Conditional cash transfer programs: Effects on growth, health and development in young children |
| Gibbs et al. | 2012 | Low | Combined structural interventions for gender equality and livelihood security: a critical review of the evidence from southern and eastern Africa and the implications for young people |
| Kavle et al. | 2018 | Low | Community-based distribution of iron-folic acid supplementation in low- and middle-income countries: A review of evidence and programme implications |
| Masino and Niño‐Zarazúa | 2015 | Low | What works to improve the quality of student learning in developing countries? |
| Ralston et al. | 2017 | Low | The impacts of safety nets in Africa: What are we learning? |
| Schenk and Michaelis | 2010 | Low | Community interventions supporting children affected by HIV in sub-Saharan Africa: A review to derive evidence-based principles for programming |
| Temidayo and Awojobi | 2020 | Low | Relationship between cash transfer programmes and school outcomes in Africa and Latin America: A systematic review |

#### 2.4 Number of reviews that scored positively across each JBI item

### Appendix 6 – Summary of findings from meta-analyses

The table presents results of meta-analyses that provided gender disaggregated findings. The review does not report on measures of effect size and confidence intervals.

| **Authors (Year)** | **Intervention category** | **Intervention** | **Sample size (No. of included studies)** | **Summary** |
| --- | --- | --- | --- | --- |
| Baird et al. (2013) | Social assistance | Conditional and unconditional cash transfer | 75 | The pooled effect sizes on enrolment for UCT and CCT among boys are OR=1.28 (95% CI = 0.97-1.69) and OR=1.55 (95% CI = 1.28-1.86), respectively. The same figures are OR=1.32 (95% CI =1.10-1.60) and OR=1.64 (95% CI =1.43-1.88) among girls. |
| Brody et al. (2015) | Labour market programmes; Social insurance | Self-help group programmes; Micro-insurance | 34 | Women’s self-help groups have a positive effect (SMD=0.18, 95% CI=0.05-0.31; evidence from 7 studies) on women’s economic empowerment. Self-help groups have a positive effect level (SMD=0.19, 95% CI=0.09-0.29; evidence from 7 studies) on women’s social empowerment. The average effect of women’s self-help groups on political empowerment is also positive (SMD=0.19, 95% CI=0.01-0.36). No impact of women’s self-help groups on psychological empowerment (SMD=0.02, 95% CI=-0.21-0.26; evidence from 2 studies). |
| Chinen et al. (2017) | Labour market programmes | Vocational and business training programmes | 35 | Vocational trainings increase the likelihood of employment and formal employment by 11% (95% CI=1.03-1.18; evidence from 8 programmes) and 8% (95% CI=1.00-1.18; evidence from 5 programmes), respectively. The average effect of vocational training on earnings was 5.54% ( 95% CI =2.50%-8.96%; evidence from 8 programmes) or 0.11 standardized mean differences ( 95% CI = 0.05-0.18; evidence from 8 programmes). Business training increased women’s sales or profits by 0.10 standardized mean differences ( 95% CI = 0.00-0.20; evidence from four programmes) or 6.83% (95% CI =0.15%-9.95%; evidence from four programmes). |
|  | Social assistance | Cash plus vocational and business training |  | Business training combined with cash transfers or life skills trainings increased the likelihood of self-employment by 73% (95% CI=1.28-2.09; evidence from three programmes). |
| Choko et al. (2018) | Social assistance | Cash transfers; vouchers | 9 | Financial incentives improved linkage to HIV treatment in three of the five trials that investigated this outcome. Significant improvements were observed among post-partum women RR=1.26 (95% CI=1.08-1.48). An overall 4-fold increase in the uptake of circumcision among HIV negative uncircumcised men with overall RR=4.00 (95% CI=2.17-7.37) was observed. |
| Dror et al. (2016) | Social insurance | Community-based health insurance | 54 | Three of nine studies in the Sub-Saharan Africa region reported a positive association between the enrolment in community-based health insurance and male-headed household, but the remaining studies reported almost zero, or highly negative association between the two. The summary effect is estimated as negative for both regions (-0.0505 for Asia,-0.3556 for Sub-Saharan Africa) and for the two regions combined (-0.359). However, the results are only indicative and not conclusive because the standard errors for the summary effect size could not be calculated. |
| Durao et al. (2020) | Social assistance | Unconditional cash transfers | 59 | A meta-analysis of two studies showed a reduction in stunting with UCTs (OR=0.62, 95% CI=0.46-0.84; 2914 children). A meta-analysis of three studies showed a small improvement in food security scores (SMD=0.18, 95% CI=0.13-0.23; 6209 households). |
| Ibanez et al. (2017) | Labour market programmes; Social care | Multiple interventions (e.g., subsidised employment; tax deductions money; in-kind grants; job placements; support for entrepreneurial activities; childcare; care for older persons) | 35 | *Interventions that aimed at women's economic empowerment have a positive and significant effect on income. Beneficiaries receive 3% standard deviations more income than non-beneficiaries (p= 0.0642). The interventions have no significant effect on the number of assets neither for on average for all interventions nor for any particular intervention (p=0.423). There were no significant effects on savings or debts. Beneficiaries are 3% standard deviations more satisfied (measures of welfare) than non-beneficiaries (p= 0.0679). |
| Kennedy et al. (2020) | Social assistance | Economic compensation interventions | 8 | Combining effect size data from four RCTs of all types of economic compensation interventions, shows that economic compensation increased the uptake of voluntary male circumcision compared with control groups that did not receive such interventions, or that received lesser forms of the interventions (RR= 5.23, 95% CI=3.13-8.76). |
| Kluve et al. (2017) | Labour market programmes | Active labour market programmes | 113 | Greater effect sizes for young women (SMD=0.08, 95% CI=0.05-0.12) compared to young men (SMD=0.06, 95% CI=0.02-0.1) across employment outcomes. Differences were also identified between young women (SMD=0.08, 95% CI=0.05-0.11) and young men (SMD=0.05, 95% CI=0.02-0.09) in earning outcomes. |
|  |  |  |  |  |
| Kristjansson et al. (2015) | Social assistance | Distribution of supplementary food, with or without added micronutrients | 32 | There were no significant subgroup differences in either the analysis for weight (Chi² = 0.06, df = 1, p-value = 0.80, I² = 0%) or height (Chi² = 0.54, df = 1, p-value = 0.46, I² = 0%). |
| Langer et al. (2018) | Social assistance; social insurance; labour market programmes; social care | Training and job placement interventions | 19 | There was an increase in women’s formal wage employment of 0.159 standardised mean difference (SMD) (95% CI=0.09-0.23, 8 studies), which translates into a 7.8% greater increase in formal wage employment for women taking part in the training and placement programmes when compared to a control group. These interventions were further effective in increasing women’s income, and a meta-analysis on income outcomes identified a positive pooled effect size of training and job placement interventions on women’s income of 0.145 SMD (95% CI=0.07-0.22). This effect size expressed a 7.2% greater increase of income for women taking part in the interventions as compared to a control group. |
| Målqvist et al. (2013) | Social assistance | Conditional cash transfer; voucher programmes | 18 | Meta-analyses of the effectiveness of incentives programs showed a pooled effect size of RR=1.66 (95% CI=1.43–1.93) for antenatal care attendance (four studies with 2,476 participants) and RR=2.37 (95% CI=1.38–4.07) for health facility delivery (five studies with 25,625 participants). Meta-analyses were not performed for any of the other outcomes due to scarcity of studies. |
| Manley et al. (2012) | Social assistance | Conditional and unconditional cash transfer | 24 | On average girls see impacts that are 0.20 height-per-age larger than boys, but results are not statistically significant. |
| Petrosino (2012) | Social assistance | Conditional and unconditional cash transfers; food transfers; vouchers; school fee reduction or elimination; scholarships or fellowships to offset the costs of schooling, in-kind transfers | 73 | Eight studies tested interventions that specifically targeted females, including six that were scholarship/fellowship programs. The average effect for female-focused interventions was slightly larger (d=0.18, 95% CI=0.06-0.30) among girls alone than among programmes targeting both girls and boys (d=0.15, 95% CI=0.11-0.19), but the there was no evidence of a difference in the two groups. |
| Snilstveit et al. (2016) | Social assistance | School feeding programmes; cash transfers; scholarships and allowances; reducing or eliminating school user fees; in-kind transfers | 420 | The results of sub-group analysis by gender suggest effects of slightly smaller magnitude for both groups on school enrolment (girls: SMD=0.07, 95% CI [0.03-0.11]; boys: SMD=0.10, 95% CI [0.05-0.16]), however the studies that report on gender sub-groups are likely not a representative sample and the results should therefore be interpreted with caution. The overall average effect of cash transfers on school dropout is -0.12, 95% CI [-0.16, -0.07]. The results from the studies with sub-group analysis by gender suggest slightly larger overall effects, especially for girls. The results for boys on school completion were similar, but less precise than the results for the full sample (SMD=0.11, 95% CI=-0.12, 0.33). The results for girls were larger in magnitude than that of the main sample (0.18 SMD, 95% CI [-0.03,0.40]), although the confidence interval is crossing the line of no effect. As for the main sample heterogeneity is high. Combining the effects for two studies on math scores for girls and boys respectively indicates a slightly smaller in magnitude and less precise than the effect for the full sample, but not substantively different from each other (girls: SMD=0.06, 95% CI [-0.09, 0.21]; boys, SMD=0.06, 95% CI [-0.03, 0.14]) |
| Tripney et al. (2013) | Labour market programmes | Technical and vocational education and training interventions | 26 | Treatment effects for female youth on paid employment, g=0.1 (95% CI [0, 0.2]), appear to be slightly larger than for male youth, g=0.01 (95% CI [-0.08, 0.09]). However, the observed differences in mean effects were not statistically significant (Qb = 2.1; p = 0.147). For female youth, the individual effect sizes were meta-analysed to produce a weighted average effect size of g=0.14 (95% CI [0.08, 0.21]) on earnings. For male youth, the pooled estimate of effect was also positive (g=0.09) and again the confidence intervals do not include zero (95% CI [0.02, 0.16]). For female youth, the individual effect sizes were meta-analysed to produce a weighted average effect size of g=0.03 (95% CI [-0.08, 0.13]) on self-employment earnings. For male youth, the pooled estimate of effect was negative (g=-0.06) although the confidence intervals cross zero (95% CI [-0.18, 0.05]). For female youth, the individual effect sizes were meta-analysed to produce a weighted average effect size of g=0.16 (95% CI [0.04, 0.28]) on hours worked. For male youth, the pooled estimate of effect was negative (g=-0.09; 95% CI [-0.2, 0.01]). The observed differences in mean effects were statistically significant (Qb = 10.1; p = 0.00151). |

### Appendix 7 – Impact of social protection programmes on gender equality outcomes

### Appendix 8 – Evidence gap maps

1. Map of reviews reporting on intervention effectiveness by intervention type

###

1. Map of reviews reporting on implementation by intervention type

1. Map of review reporting on design features by intervention type

1. Map of reviews reporting on implementation features by outcome area

1. Map of reviews reporting design features by outcome area
